# A Synovium‐on‐Chip Platform to Study Multicellular Interactions in Arthritis

**DOI:** 10.1002/adhm.202504857

**Published:** 2026-01-24

**Authors:** Laurens R. Spoelstra, Fleur R. Semmekrot, Nuno Araújo‐Gomes, Daniël Wijnperle, Monique M. A. Helsen, Martijn H.J. Van Den Bosch, Loes. I. Segerink, Séverine Le Gac, Marcel Karperien

**Affiliations:** ^1^ Department of Developmental BioEngineering Technical Medical Centre University of Twente Enschede The Netherlands; ^2^ BIOS Lab on a Chip group MESA+ Institute for Nanotechnology Technical Medical Centre University of Twente Enschede The Netherlands; ^3^ Applied Microfluidics for BioEngineering Research MESA+ Institute for Nanotechnology Technical Medical Centre University of Twente Enschede The Netherlands; ^4^ Experimental Rheumatology Radboud University Medical Center Nijmegen The Netherlands

**Keywords:** arthritis, cellular crosstalk, endothelial remodeling, fibroblast‐like synoviocytes, organ‐on‐chip, synovium‐on‐chip

## Abstract

Arthritis progression is modulated by the synovium, yet its cellular crosstalk remains poorly understood, partly due to the limited availability of human‐relevant preclinical models. Therefore, we developed a compartmentalized synovium‐on‐chip (SoC) with a co‐culture of primary human fibroblast‐like synoviocytes (FLS), THP‐1‐derived macrophages, and endothelial cells across a 2‐µm‐thick microporous polydimethylsiloxane membrane. This microscale architecture sustains the co‐culture of all cell types for at least ten days, preserving lineage‐specific markers (cadherin‐11, CD163, VE‐cadherin). Without external cues, endothelial lumen remodeling was triggered by FLS migration through the membrane pores. Machine‐learning‐based image analysis revealed pronounced endothelial phenotypic shifts in response to FLS, highlighting the importance of intercellular communication on the cellular scale. Upon stimulation with TNF‐α, synovial inflammation was successfully established, with robust cytokine upregulation. By capturing dynamic, migration‐driven interactions between synovium and vasculature in vitro, our SoC platform provides a powerful tool to further study the mechanisms of arthritis progression in the synovium and to identify new targets for therapeutics development.

## Graphical abstract

The Synovium‐on‐Chip comprises a thin microporous PDMS membrane to support co‐culture of fibroblast‐like synoviocytes (FLS), THP‐1‐derived macrophages, and endothelial cells, enabling real‐time analysis of synovial‐vascular interactions. FLS migration through the pores drives endothelial remodeling, while TNF‐α stimulation induces robust inflammatory activation, providing a versatile platform for studying mechanisms of arthritis progression.

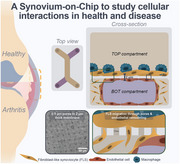

## 1 Introduction

**FIGURE 1:**
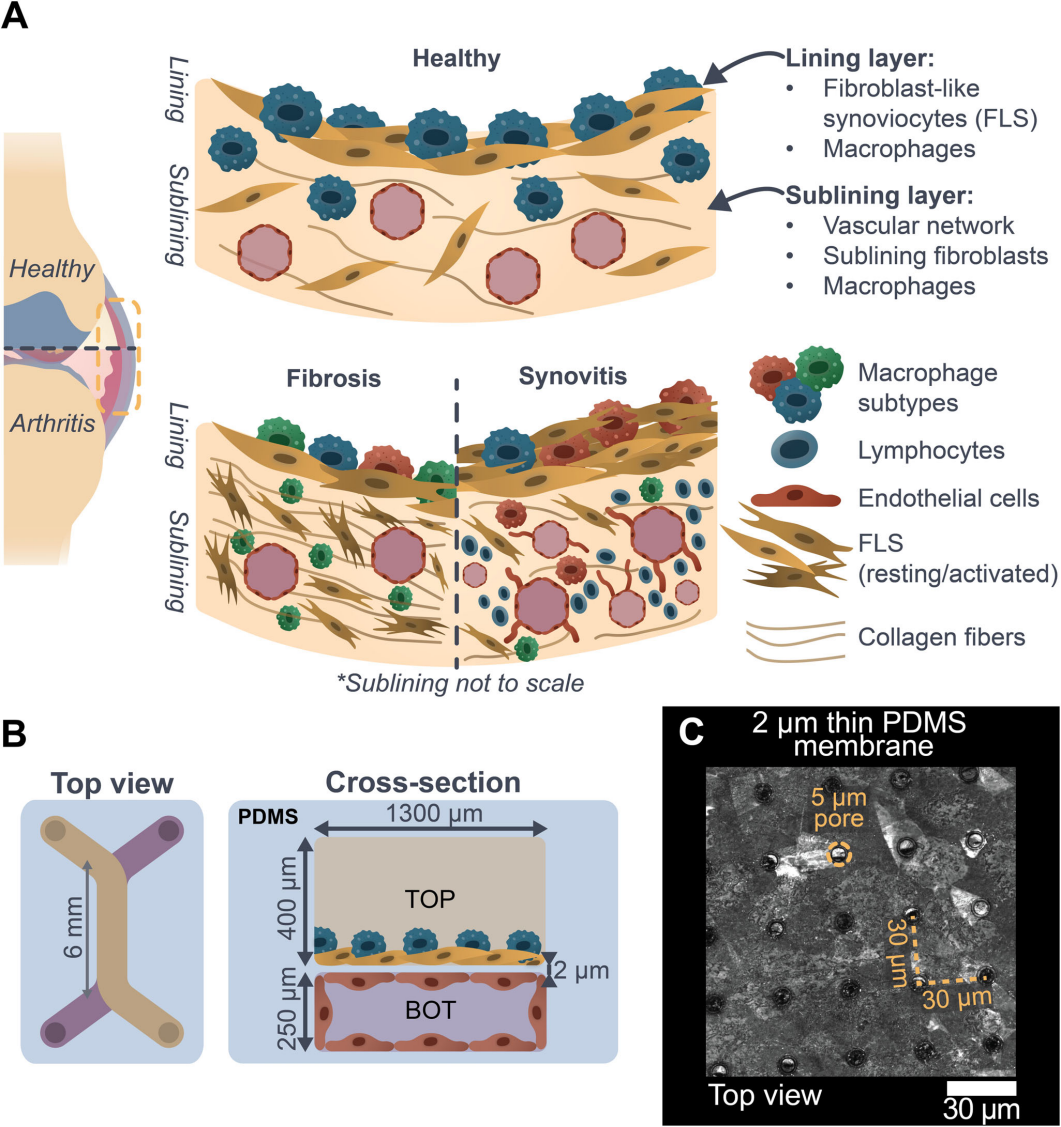
Biomimetic synovium‐on‐chip (SoC). A) *Top*: healthy synovium consisting of a (i) lining layer with 1–3 layers of FLS and resident macrophages and (ii) a vascularized sublayer with sublining FLS and various immune cells [1, 2]. *Bottom*: fibrotic and inflammatory (synovitis) subtypes commonly found in arthritis, characterized by changes in cell‐cell interactions, cell phenotypes, and behavior. Fibrosis: FLS activation, excessive matrix deposition, and imbalanced macrophage phenotypes. Synovitis: FLS activation, pro‐inflammatory macrophage imbalance, angiogenesis, lining hyperplasia, and immune cell infiltration. Illustration based on references [3, 9, 19]. B) Schematic overview of the SoC device. The full‐PDMS chip features two culture chambers (TOP and BOT) separated by a 2‐µm‐thin, porous PDMS membrane (C) to mimic the in vivo architecture of the synovium closely. TOP chamber: patient‐derived FLS and THP‐1‐derived macrophages as a model for resident synovial macrophages. BOT chamber: RFP‐HUVECs covering all surfaces to mimic sublining vasculature. The design features a top layer with 3‐mm‐diameter reservoirs (Figure S1A), that allows full media separation between the TOP and BOT chambers. C) Confocal reflection microscopy image of the 2‐µm‐porous PDMS membrane that separates the TOP and BOT compartments [27, 28]. The 5‐µm diameter circular pores, spaced 30 µm apart, enable cell‐cell contacts and cell migration.

The synovial membrane plays a key role in diarthrodial joints, such as the knee [1]. In the healthy knee joint, the synovial membrane ensures joint homeostasis and lubrication by the production of synovial fluid [1, 2]. Also known as the synovium, this specialized connective tissue covers the inner side of the joint capsule and can be subdivided into a lining and a sublining layer [1, 3]. The lining layer comprises several cell layers of fibroblast‐like synoviocytes (FLS) and resident macrophages that clear up cellular debris and modulate inflammation in the joint [1, 2, 3]. The sublining layer consists of a vascularized collagenous matrix with scattered FLS, adipocytes, macrophages, and various other immune cells (Figure 1A) [1, 3].

The synovium is also involved in the pathogenesis of whole‐joint arthritic diseases such as osteoarthritis (OA) and rheumatoid arthritis (RA) [4, 5, 6, 7], which altogether affect millions of people globally [5, 7]. While OA and RA have different pathologies, they both cause disruptions in synovial membrane biology. In RA and a subset of patients with OA, synovial fibrosis occurs, characterized by excessive extracellular matrix (ECM) deposition in the sublining layer, eventually leading to joint stiffness and pain (Figure 1A) [8, 9, 10, 11, 12]. In both OA and RA, synovial inflammation (synovitis) leads to FLS activation and migration, disrupting normal cellular communication, enhancing production of cytokines and growth factors, and increasing the release of matrix‐degrading enzymes. Synovitis furthermore stimulates neo‐angiogenesis, an imbalance between pro‐ and anti‐inflammatory macrophage populations, and immune cell infiltration from the nearby vasculature (Figure 1A) [5, 6, 7, 8, 13, 14, 15, 16, 17, 18, 19, 20].

To date, there is a lack of adequate models to emulate the role of the synovial membrane in healthy and disease states [21, 22, 23, 24]. Traditionally, either animal models, which lack physiological relevance, or oversimplified in vitro models have been employed [21, 22, 23, 24]. Of interest is the work of Broeren et al., who, by using synovial organoids generated from RA biopsies, successfully modeled the lining and sublining layers of the synovium and recreated, to a certain extent, fibrosis and hyperplasia [25]. However, that model did not include the vascular features of the synovium, which are required to recapitulate monocyte infiltration. In another study, a 2D cell culture of FLS was used to examine their response to stimulation with pro‐inflammatory cytokines like tumor necrosis factor‐alpha (TNF‐α) [26]. While that work recapitulated the increased proliferation of FLS upon inflammatory stimulation, it lacked physiological complexity since it only included FLS.

To better emulate the physiological complexity of human tissues, organ‐on‐chip models (OoCs) have emerged as a promising alternative to conventional in vitro models [27, 28]. OoCs are micro‐engineered models of human tissues that provide the unique feature to control (bio)mechanical and chemical stimulation and the cellular microenvironment [27, 28]. To date, a handful of synovium‐on‐chip (SoC) models have been reported [29, 30, 31, 32, 33]. For example, Mondadori et al. [30] studied monocyte extravasation and the interactions between FLS, chondrocytes, and endothelial cells in the presence of OA patient synovial fluid. They used an organ‐on‐chip model with hydrogel components for the synovium and cartilage tissue mimics separated by an endothelial channel and a synovial fluid channel. However, this model lacks the structured organization of the synovial membrane into an intima and subintima. More recently, Thompson et al. [29]. reported an SoC model based on a commercially available two‐compartment chip with an integrated 50‐µm‐thick microporous polydimethylsiloxane (PDMS) membrane that can be stretched on demand. This model features an endothelial compartment as well as healthy donor FLS and was used to investigate both the release of inflammatory mediators and monocyte recruitment in response to mechanical stretching and/or inflammatory stimulation (IL‐1β). However, the use of a relatively thick membrane (50 µm) is expected to hamper direct cell‐cell communication and cell migration between the compartments. Furthermore, both these models are missing a resident immune component. This gap was recently addressed by Lee et al. [33], with a SoC model incorporating a vascular compartment separated by a collagen‐alginate hydrogel from a synovial lining layer. This lining layer consists of human FLS and monocyte‐derived M2c macrophages that share characteristics with in vivo macrophage‐like synoviocytes. In their work, they demonstrate that cellular communication between these macrophages and FLS is crucial for maintaining a functional lining while ensuring physiological relevance. However, FLS migration was physically limited by the hydrogel, and interactions between FLS and the vascular compartment relevant to neo‐angiogenesis were not studied. Altogether, existing models of the synovial membrane often omit a resident immune component and fail to fully cover or study the complex cell‐cell interactions between FLS, macrophages, and endothelial cells in a controllable manner and at a relevant scale.

To address this challenge, we present a full‐PDMS two‐compartment SoC platform featuring a unique 2 µm‐thick microporous PDMS membrane with 5‐µm circular pores supporting chemical communication as well as cell migration and interaction at a scale similar to the cells [34, 35]. The present work focuses on establishing the platform and its underlying methodology, which altogether provides the foundation for more in‐depth mechanistic studies of arthritis in the future. To recreate key features of the synovial lining, human donor‐derived FLS and THP‐1‐derived macrophages, serving as a model for resident synovial macrophages while establishing the platform, were co‐cultured within one compartment, while human umbilical vein endothelial cells (HUVECs) were seeded in the other compartment to mimic the synovial vasculature in the sublining. We first demonstrated successful integration of these three cell types, their inter‐compartment migration in this triple co‐culture setting, cellular phenotypic shifts, as well as remodeling of the vasculature as a result of cellular interactions and migration. Next, we stimulated the SoC using TNF‐α to induce arthritis‐like conditions and verified the subsequent release of inflammatory cytokines. Our SoC model exhibits cellular crosstalk, migration of FLS, and sprouting of endothelial cells, which are all well‐known processes implicated in arthritis. As such, we propose our SoC platform as a powerful model to further elucidate this complex cellular crosstalk in future research to deepen our understanding of cell‐cell interactions in arthritis and identify therapeutic targets for drug development.

## 2 Results

### 2.1 Development and Optimization of the SoC Platform with a Triple Co‐Culture Model

To model and study the synovial membrane in both healthy and arthritic conditions (Figure 1A), we designed a SoC platform (Figure 1B) featuring two overlaid culture compartments (TOP and BOT, *ca*. 6.0 mm long and 1.3 mm wide) separated by a horizontal 2‐µm‐thin microporous membrane (5 µm pores, 30 µm spacing, Figure 1C). The platform additionally includes a reservoir layer (3‐mm‐diameter × 3‐mm‐height cylindrical reservoirs) placed on top of the chip to ensure both complete media separation between the TOP and BOT channels as well as sufficient nutrient delivery for prolonged cell culture. The channel design was optimized for homogenous cell seeding, and the fabrication protocol was adapted, building upon previous work on similar PDMS membranes [27, 28], for wafer‐scale production of the SoC devices (up to 20 chips), while reducing the number of manual alignment steps (Figure S1).

**FIGURE 2:**
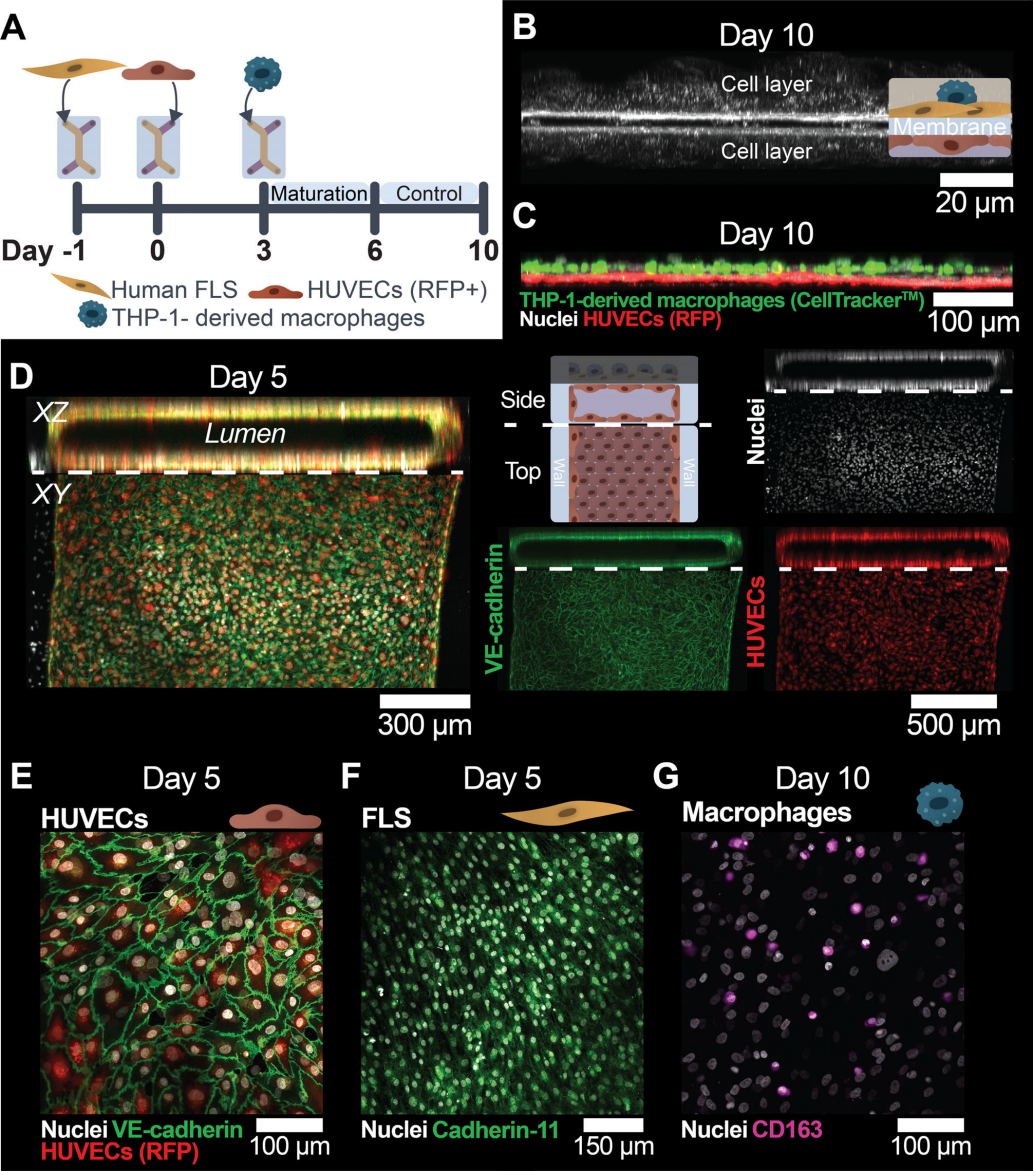
Establishment of a triple co‐culture of FLS, THP‐1‐derived macrophages, and RFP‐HUVECs in the SoC device. A) Experimental design and associated timeline in the SoC model. Devices were coated with polydopamine followed by collagen‐1 on Day ‐1, and FLS were seeded in the TOP chamber. The next day, RFP‐HUVECs were seeded to line all surfaces of the BOT chamber. After 3 days of culture, THP‐1‐derived macrophages were seeded in the TOP chamber and allowed to adhere and rest before experiments started at Day 6. B) Orthogonal projection of a confocal reflection microscopy z‐stack showing the cell layers on both sides of the thin membrane. C) CellTracker‐labeled THP‐1‐derived macrophages remained attached until Day 10 on the TOP side of the membrane, and RFP‐HUVECs remained attached on the BOT side. D) Orthogonal projections of confocal z‐stacks of the SoC model. By Day 5 (end of the maturation period), the endothelium formed a rectangular lumen in the BOT chamber. E–G) Representative images of all cell types in the SoC model. E) On Day 5, HUVECs displayed their characteristic cobblestone morphology with distinct VE‐cadherin expression at the cell borders. Note that there are more nuclei visible than there are endothelial cells, which correspond to the FLS/macrophages on the other side of the membrane. F) FLS expressed cadherin‐11 and formed a loosely connected network. G) THP‐1‐derived macrophages still adhered at Day 10 and expressed CD163, which is a characteristic macrophage marker. CD163‐negative nuclei correspond to the FLS in the TOP chamber. [Correction added on 19 March 2026 Figure 2 has been replaced.]

We next optimized the cell seeding procedures to establish a triple co‐culture with FLS, HUVECs, and a resident macrophage component. We characterized the integration of arthritis patient‐derived FLS and THP‐1‐derived macrophages seeded in the TOP channel, with HUVECs in the BOT channel (Figures 1B and 2A). The seeding density for FLS was chosen to be 2·10^6^ cells mL^−1^, which gave a confluent monolayer on Day 0. Similarly, a HUVEC seeding density of 4.5–6.0·10^6^ cells mL^−1^ resulted in a tight monolayer on the membrane on Day 2, whilst preventing cell clumps and excessive cell death. As an immune component, the THP‐1 monocytic cell line [36] was chosen, in a first instance, to develop and optimize the triple co‐culture model and underlying co‐culture protocol. THP‐1 cells are commonly used as an alternative for peripheral blood mononuclear cells (PBMCs) and can be differentiated into macrophages using well‐established procedures based on phorbol 12‐myristate 13‐acetate (PMA) [37, 38]. The seeding density of THP‐1‐derived macrophages was optimized to be 8.5–9.0·10^5^ cells mL^−1^ to ensure their homogeneous distribution on the FLS monolayer with little clump formation. These optimized cell seeding densities correspond to a theoretical initial macrophage: FLS ratio of 1:2, but in practice, this ratio is likely to be closer to 1:3, as not all seeded macrophages adhered to the FLS layer upon seeding.

After polydopamine and collagen‐1 coating to promote cell adhesion, all cell types were cultured for up to ten days in the SoC devices. First, FLS were seeded in the TOP channel on the membrane on Day ‐1 (Figures 1B and 2A) to establish the first part of the synovial lining (Figure 1A). Subsequently, on Day 0, HUVECs were introduced on the floor of the BOT chamber, as well as on the bottom side of the membrane (Figures 1B and 2A) to form an endothelial lumen mimicking the synovial sublining (Figure 1A). Finally, THP‐1‐derived macrophages were injected in the TOP chamber on Day 3 to finalize the synovial lining (Figures 1A,B and 2A), while switching the medium in this TOP compartment to THP‐1 culture medium to preserve their viability. Label‐free confocal reflection microscopy revealed that cell layers were present on both sides of the membrane, suggesting successful integration of the different cell types (Figure 2B). Next, we examined the specific distribution of all three cell types across the membrane by confocal microscopy (Figure 2C). THP‐1‐derived macrophages, labeled with CellTracker green, integrated well on top of the FLS (nuclear stain only) and remained attached on the top side of the membrane until Day 10, while the red fluorescent protein (RFP)‐expressing HUVECs localized in the BOT compartment under the membrane.

Next, we further characterized the endothelial lumen formation in the BOT channel of the SoC (Figures 1B and 2D). HUVECs formed a rectangular lumen from Day 2 onward, following the geometry of the channel, exhibiting tightly packed cobblestone‐like cells and clear cell‐cell junctions as demonstrated by the expression of VE‐cadherin at the cell‐cell junctions on Day 5 (Figure 2D,E), thereby confirming the creation of an endothelial barrier within the maturation period (until Day 6, Figure 2A). Finally, we confirmed the expression of markers characteristic of a healthy synovium in vivo by both FLS and THP‐1‐derived macrophages by immunofluorescence and confocal microscopy (Figure 2F,G). FLS expressed cadherin‐11, a characteristic marker which is important for synovial lining formation [17, 39] (Figure 2F), while THP‐1‐derived macrophages expressed the macrophage marker cluster of differentiation 163 (CD163) on Day 10 (Figure 2G). Altogether, these results confirm the establishment of a triple co‐culture model in our SoC platform within ten days, including an endothelial lining with junctional VE‐cadherin expression and a resident macrophage component.

### 2.2 FLS Migration‐Induced Spontaneous Endothelial Lumen Remodeling in the SoC Platform

**FIGURE 3:**
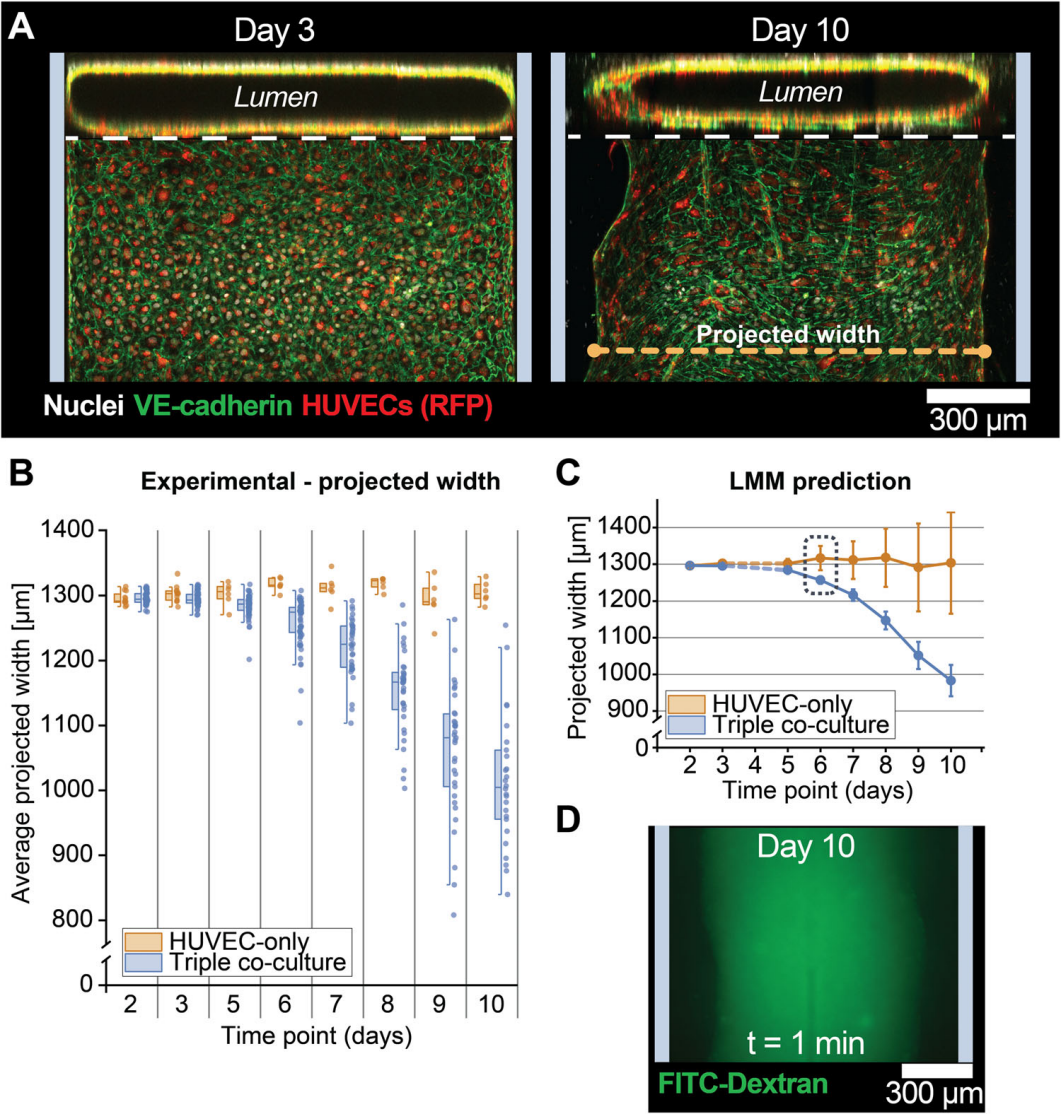
Progressive remodeling of the endothelial lumen in the SoC. A) Orthogonal views of confocal z‐stacks of SoC models stained for VE‐cadherin. The lumen width decreased between Day 3 and Day 10, with the lumen detaching from the walls (gray banners). VE‐cadherin expression was preserved, indicating that the endothelial cells remained tightly connected. B) Evaluation of the projected lumen width (definition in (A)) over the culture period for HUVEC‐only controls (orange) and triple co‐culture SoC models (blue) (see Figure S3 for details on data analysis). Each data point represents one chip. Number of samples, each depicted as HUVEC‐only (orange)/triple co‐culture (blue): N = 12/68 (Day 2), N = 13/68 (Day 3), N = 6/65 (Day 5), N = 6/62 (Day 6), N = 6/34 (Day 7), N = 6/34 (Day 8), N = 6/33 (Day 9), N = 6/26 (Day 10). C) Linear Mixed Model predictions of projected width for HUVEC only (orange) and triple co‐culture (blue). Error bars: 95% confidence interval. D) The endothelial lumens were perfusable as indicated by the 4‐kDa FITC‐dextran fluorescence within the lumen (channel walls indicated with gray banners for reference). After a few minutes, the FITC‐dextran started to leak out into the space surrounding the lumen (Figure S2).

Interestingly, HUVECs in the bottom chamber detached from the channel walls in the triple co‐culture experiments from Day 5 onward (Figure 3) while forming a lumen structure as confirmed by 3D confocal microscopy of VE‐cadherin‐stained devices on Days 3 (before remodeling onset) and 10 (Figure 3A). HUVECs started forming an oval‐shaped lumen while retaining junctional VE‐cadherin expression, suggesting that the endothelial lining remained intact. To assess the reproducibility and progression of this remodeling, we performed four independent experimental runs, in which we systematically measured the projected lumen width from the RFP channel images during the culture period, for either the established triple co‐culture model or a device only comprising an endothelium in the bottom compartment as a control (HUVEC‐only). For the latter controls (Figure 3B), the average projected width remained constant throughout the entire culture period, roughly equaling the channel width of 1300 µm (Day 2: 1295.42 ± 9.77 µm (±std), Day 10: 1304.92 ± 16.71 µm; note that due to limited pixel resolution, slight edge effects during imaging, and migration of endothelial cells to the TOP compartment in the absence of FLS, this value exceeds the width of the channel). In contrast, for the triple co‐culture, while the initial projected width on Day 2 (1296.04 ± 8.76 µm) was in line with the controls, it progressively decreased to 1284.32 ± 16.50 µm on Day 5 and even to 1023.11 ± 99.76 µm on Day 10. A linear mixed model analysis (Figure 3C) revealed a statistically significant difference between the HUVEC‐only controls and the triple co‐culture groups from Day 6 onward, where the 95% confidence intervals (CI) stopped overlapping (HUVEC‐only: {1283.53 µm–1394.714 µm}, triple co‐culture: {1247.35 µm–1276.41 µm}). Note that the CI for the HUVEC‐only controls is relatively large, which might be impacted by the low number of samples considered in this condition (N = 6 (Day 10) vs. N = 13 (Day 2)) compared to the triple co‐culture (N = 26 (Day 10) vs. N = 68 (Day 2)). Finally, we verified the barrier function of the endothelial lumens with a 4 kDa FITC‐dextran probe on Day 10. Initially, the lumens retained the FITC‐Dextran probe (Figure 3D), confirming the barrier formation, but after a few minutes, the dye started to leak out into the surrounding, as expected considering the low molecular weight (MW) of the employed FITC Dextran probe (Figure S2).

**FIGURE 4:**
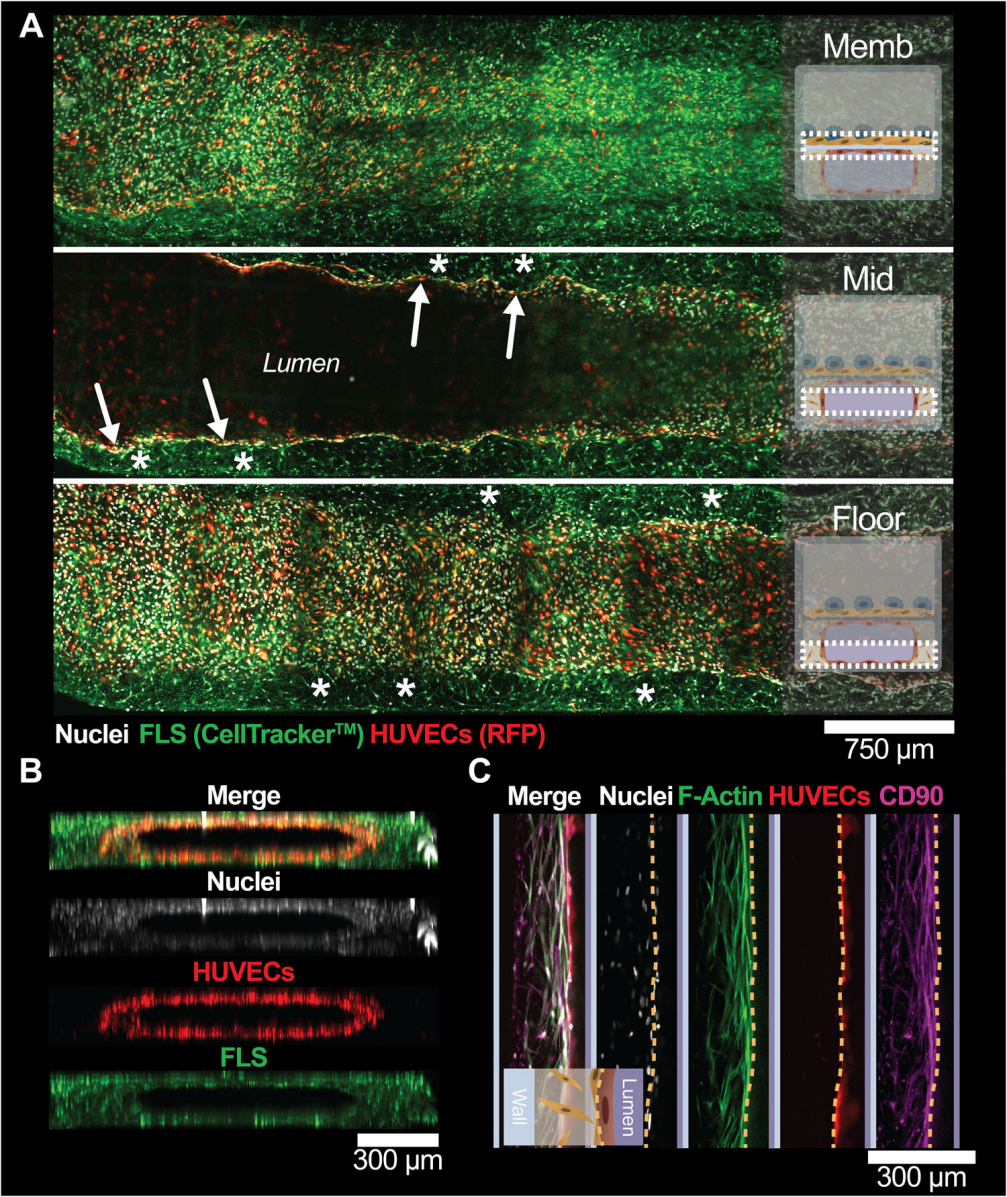
FLS that migrated through the porous membrane surrounded the endothelial lumen. A) Confocal microscopy tile scan images of the BOT chamber of SoC triple co‐culture samples with CellTracker‐labeled FLS taken at the membrane layer (Memb, *top*), middle‐height of the chamber (Mid, *middle*), and bottom of the chamber (Floor, *bottom*). CellTracker‐labeled FLS (examples indicated with asterisks) surrounded the endothelial lumen edge (arrows) and migrated throughout the BOT chamber to the floor of the chip. B) XZ orthogonal projections of a confocal microscopy z‐stack showing the FLS surrounding the RFP‐expressing endothelial lumen (red) in the BOT chamber. C) Confocal microscopy images of the extraluminal space next to the channel walls. Yellow dashed lines indicate the endothelial lumen edge. FLS have filled the extraluminal space (green) and these cells express CD90 (magenta). All images were taken on Day 10.

Given the known migratory nature of FLS derived from arthritic joints [15, 40], we hypothesized that FLS migrated through the porous membrane to surround the endothelial lumen, causing tissue remodeling in the bottom compartment. To test this hypothesis, FLS were labeled with CellTracker green to track them over time in the devices. On Day 10, 3D confocal microscopy confirmed the redistribution of the FLS (Figure 4A,B): they were found throughout the BOT chamber, not only on the membrane side, but also on the floor of the channel, as well as in the extraluminal space around the endothelium at half‐height across the full length of the device (Figure 4A, see white asterisk annotation). Altogether, the FLS surrounded the RFP‐expressing HUVEC‐based lumen, inhabiting the extraluminal space. This apparent self‐organization of FLS and endothelial cells is consistent with previous findings in synovial histology sections [17] and when using FLS‐HUVEC co‐culture organoids [41] that both showed FLS surrounding HUVEC tubular structures. Furthermore, we confirmed that the FLS expressed cluster of differentiation 90 (CD90, Figure 4C), in line with previous findings in the synovium and aforementioned organoids [17, 41]. Taken together, these data confirm that FLS migrated through the 5‐µm‐sized pores in the membrane to colonize the bottom chamber, which is likely why endothelial remodeling occurs in our SoC devices.

### 2.3 Endothelial Cell Migration and Phenotypic Shift in Response to FLS

**FIGURE 5:**
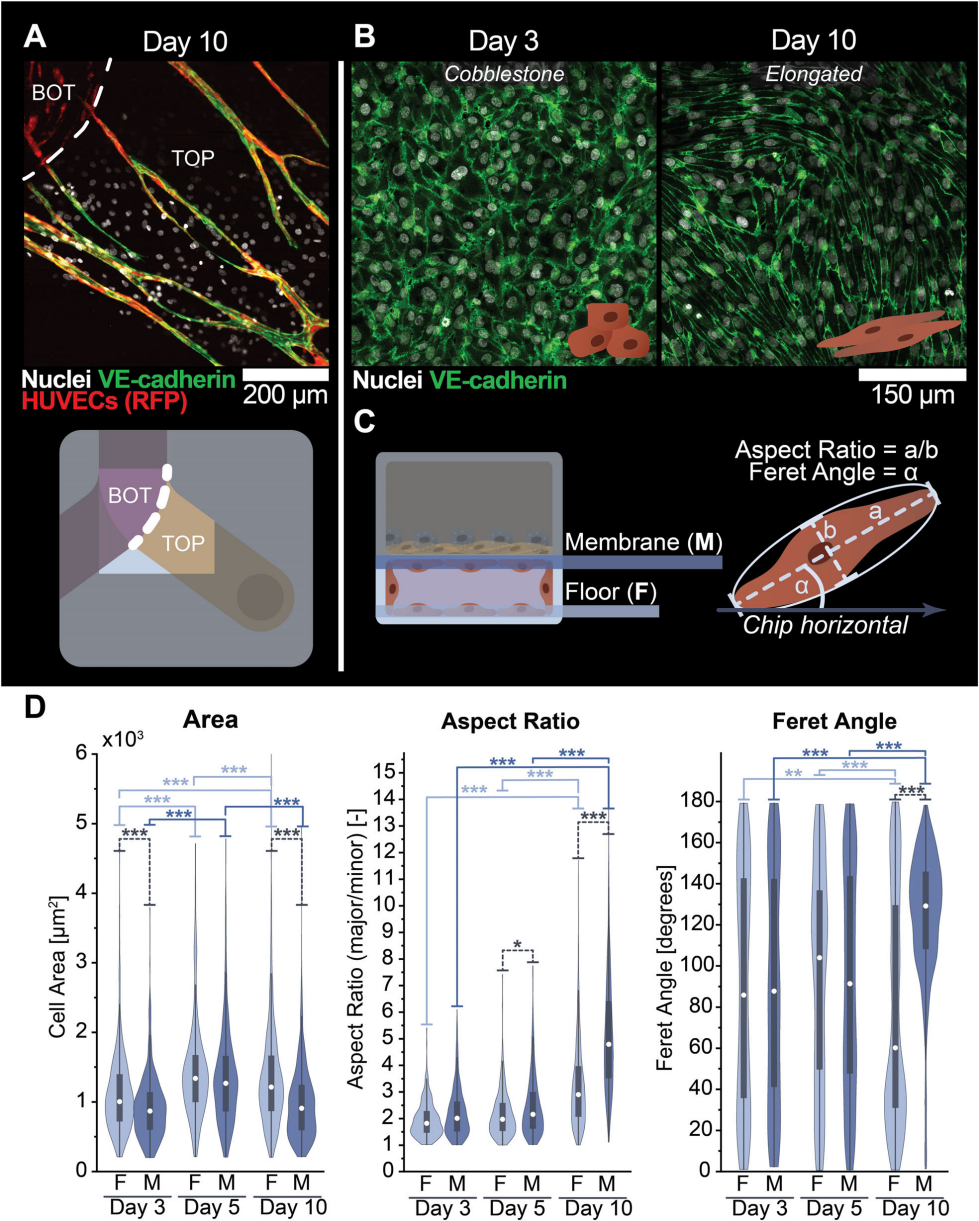
Angiogenic sprouting and endothelial morphology shift. A) Confocal microscopy image of the interface (white dashed line) between the TOP and BOT compartments (see schematic below) showing RFP‐ and VE‐cadherin‐positive endothelial sprouts in the inlet channel of the TOP chamber. B) VE‐cadherin staining on Days 3 and 10 revealed a clear morphology shift from a typical cobblestone‐like to a more elongated morphology with retained VE‐cadherin expression. C) *Left*: definition of the floor (F) and membrane (M) locations in the BOT chamber for quantitative analysis of the cell morphology and orientation. *Right*: schematic illustration of the aspect ratio and Feret angle shape descriptors. Chip horizontal based on orientation in (A). D) Cellpose machine‐learning‐assisted analysis of the cell area, aspect ratio, and Feret angle of HUVECs in the SoC. Number of cells per distribution, denoted as Floor/Membrane: N = 344/399 (Day 3), N = 410/521 (Day 5), N = 1984/1554 (Day 10). Statistical significance was tested with a Kruskal‐Wallis ANOVA with Dunn's post‐hoc test with significance levels set at *p* < 0.05. Significance indicated as: ^*^ *p* < 0.05, ^**^ *p* < 0.01, ^***^ *p* < 0.001.

In parallel to the endothelial lumen remodeling, VE‐cadherin‐expressing cells originating from the BOT compartment were found in the TOP compartment in a subset of the triple co‐culture models (Figure 5A; Figure S4A), suggesting the simultaneous occurrence of angiogenic sprouting. Triggered by this observation, we further examined the endothelial cell morphology and their VE‐cadherin expression in the BOT compartment. Interestingly, while endothelial cells displayed a traditional cobblestone‐like morphology on Day 3, they exhibited a more elongated morphology on Day 10 (Figure 5B). Despite these morphological changes, VE‐cadherin expression was preserved, suggesting that the endothelial characteristics and barrier function were maintained throughout the culture period. This unexpected morphological change was further characterized quantitatively in terms of cell area, aspect ratio, and Feret angle by machine‐learning‐assisted cell segmentation using Cellpose [42, 43, 44]. For segmentation, we used VE‐cadherin confocal microscopy images on the channel floor and membrane areas of the BOT compartment on Days 3, 5, and 10 (Figure 5C). This analysis revealed significant changes in these shape descriptors during the culture (Figure 5D). Initially, the cell area increased from Day 3 to Day 5 for both the floor and membrane‐attached cells (all *p* < 0.001), followed by a return to the Day 3 values for the membrane‐attached cells (p>0.05) on Day 10. Conversely, the cell surface area of the floor‐attached cells remained elevated compared to Day 3 (*p* < 0.001). The aspect ratio was not significantly different between the two locations in the BOT compartment on Day 3 (medians: floor 1.82, membrane 2.01; p>0.05) but was slightly elevated on the membrane compared to the floor on Day 5 (medians: floor 1.98, membrane 2.16; *p* < 0.05). On Day 10, the cell aspect ratio was significantly elevated on the membrane compared to the floor‐attached cells (medians: floor 2.90, membrane 4.79; *p* < 0.001). For both locations in the device, the aspect ratio continuously increased from Day 3 to Day 10 (all *p* < 0.001). This elongation of endothelial cells in vitro in the SoC devices, yet in the absence of dynamically applied flow, is in line with previous reports describing the response of endothelial cells to vascular endothelial growth factor (VEGF) [45], which is expected to be released by FLS in our model, as previously reported in direct co‐culture with fibroblasts [46]. In addition, the elongated endothelial cells oriented themselves at approximately 60 (floor) or 130 degrees (membrane) relative to the horizontal axis of the chip (as defined in Figure 1B) on Day 10. Remarkably, there was no preferred orientation on Days 3 and 5 (all comparisons p>0.05). Of note, all phenotypic changes were absent in HUVEC monocultures in the SoC devices (Figure S4B), while lumen remodeling was observed in co‐cultures of FLS and HUVECs in the SoC devices in the absence of THP‐1‐derived macrophages in preliminary experiments (Figure S5). Altogether, these phenotypical shifts and the lumen remodeling between Days 3 and 10 suggest dynamic changes in the endothelial layer and synovium model, likely as a result of the co‐culture, intercellular interactions, and cellular migration in the SoC device.

### 2.4 TNF‐α Stimulation Induced the Release of Soluble Inflammatory Mediators

**FIGURE 6:**
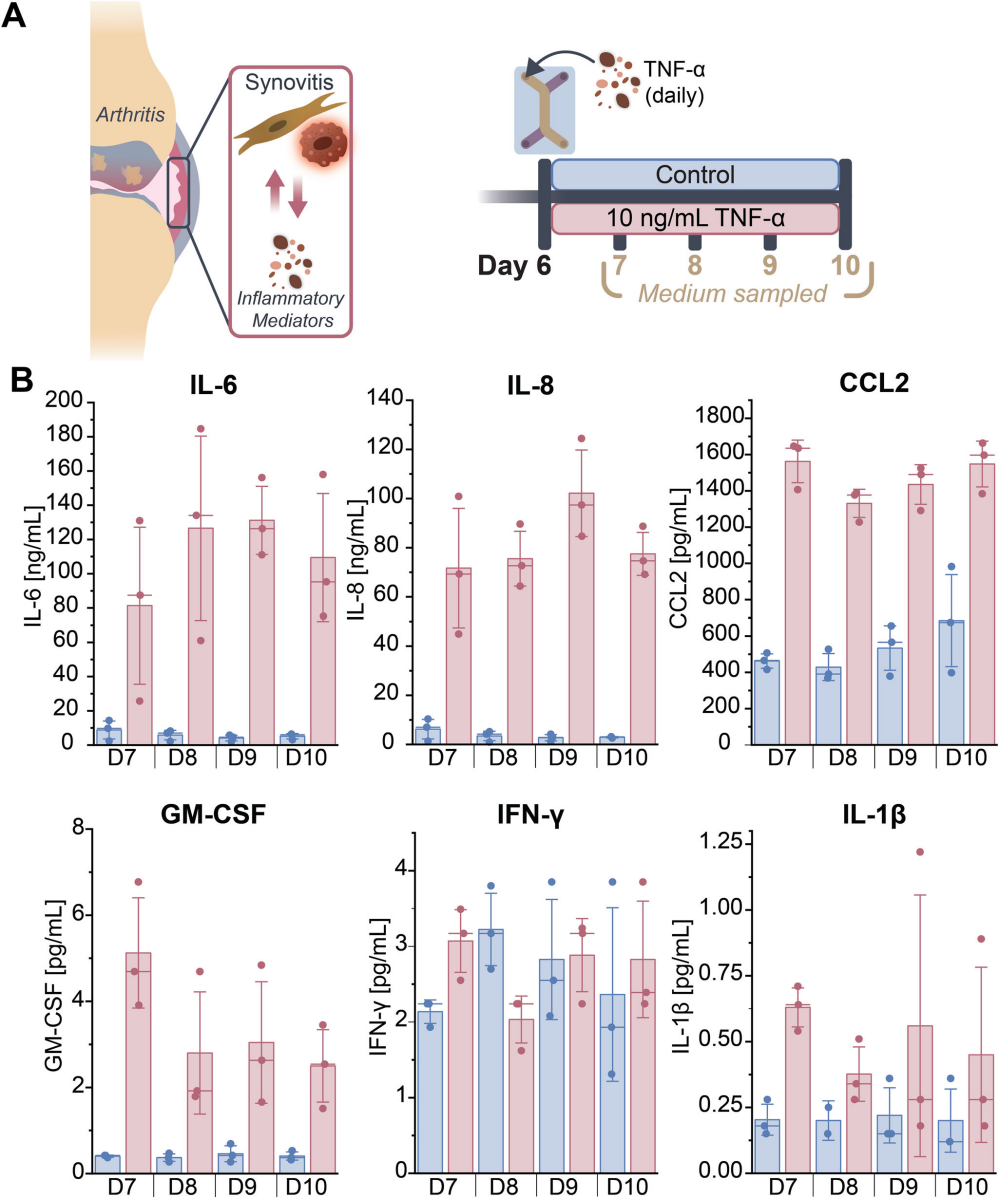
TNF‐α stimulation resulted in the secretion of inflammatory mediators in the SoC model. A) Experimental design for studying the release of inflammatory mediators in the context of synovitis. SoC devices were pre‐cultured up until Day 6 as shown in Figure 2A. From Day 6 onward, 10 ng mL^−1^ TNF‐α was added to the TOP compartment and refreshed every 24 h, while control samples received regular THP‐1 medium. Medium samples were collected from the TOP compartment on Days 7–10, daily. B) Luminex results for the release of IL‐6, IL‐8, CCL2, GM‐CSF, IFN‐γ, and IL‐1β. Each data point represents an independent experimental run, bars represent the mean, horizontal lines the median, and error bars ± 1.5 SE.

Having established a triple co‐culture model in the SoC with a resident immune component, we next investigated the response of this SoC model to inflammatory conditions as a surrogate for synovitis in vitro. To this end, SoC models were cultured as described above (Figure 2A) while being stimulated daily with 10 ng mL^−1^ TNF‐α added to the TOP compartment from Day 6 onward. Medium from the TOP compartment was collected on Days 7 to 10 (Figure 6A) and analyzed using a routine Luminex assay for IL‐6, IL‐8, IL‐1β, CCL2, GM‐CSF, and IFN‐γ secretion to get a general overview of the inflammatory status in the SoC model. This analysis revealed a clear upregulation of IL‐6 and IL‐8, CCL2, and GM‐CSF under inflammatory conditions, compared to control (Figure 6B). Of note, these cytokines, while not being specific to arthritis, are highly involved in the pathogenesis of both OA and RA [7, 8, 47]. For example, CCL2 enhances monocyte recruitment and differentiation in vivo and has been correlated to the progression of OA [47, 48]. Interestingly, no time‐dependent trend was observed for IL‐6, IL‐8, and CCL2 expression, of which the expression remained ca. 6‐10x higher (IL‐6, IL‐8) or ca. 3x higher (CCL2) in TNF‐α‐treated models compared to the controls (untreated models). Finally, for IFN‐γ, no clear correlation with TNF‐α‐stimulation was found, and the IL‐1β expression levels were overall low. Altogether, a functional inflammatory phenotype indirectly linked to arthritis was successfully established, validating our SoC model for studying synovitis in future work.

### 2.5 TNF‐α Stimulation Altered the ICAM‐1 Expression Pattern in the SoC Models

**FIGURE 7:**
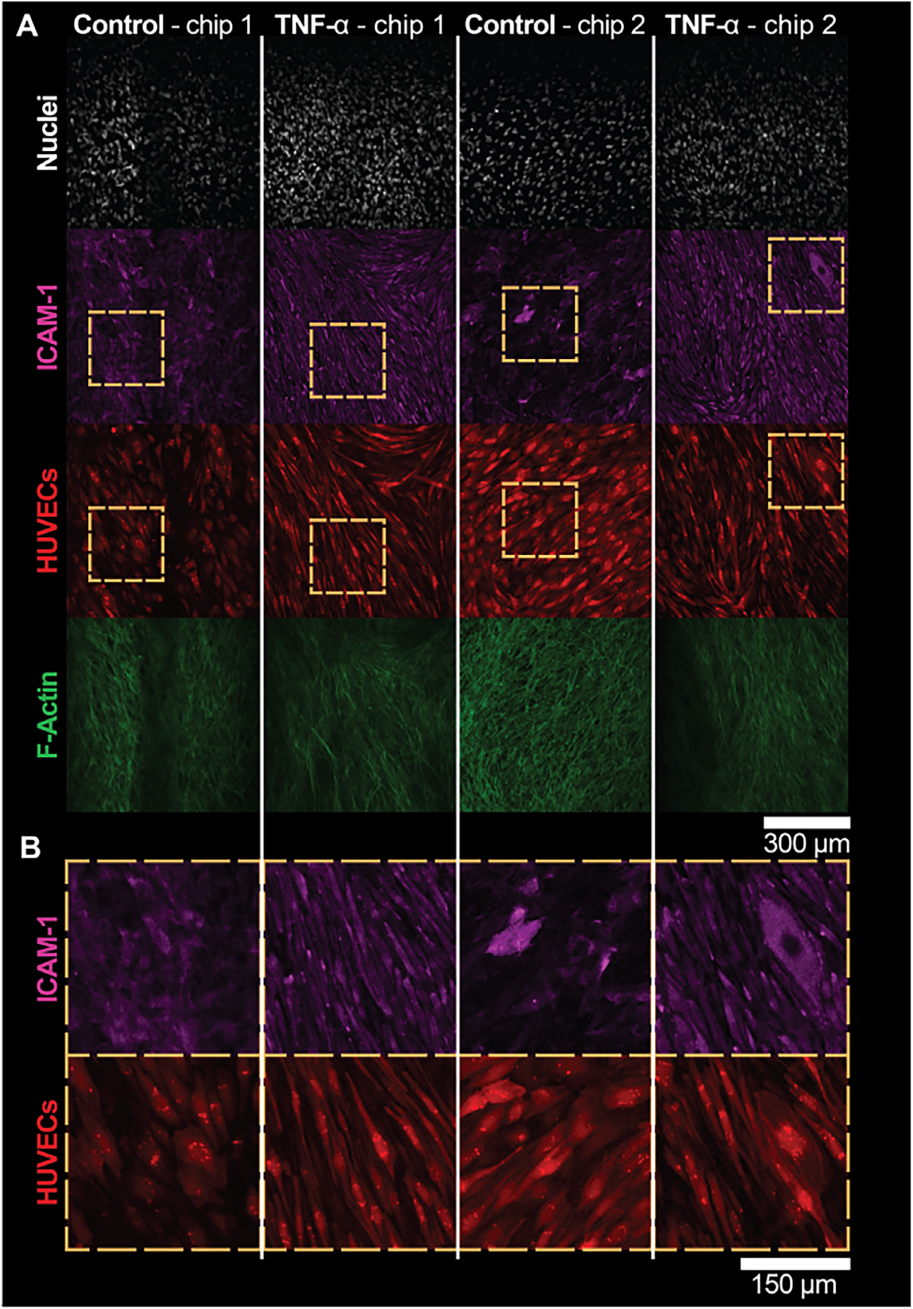
ICAM‐1 expression was upregulated by HUVECs after TNF‐α stimulation in the SoC model. A) Representative confocal microscopy images of the membrane inside the SoC device on Day 10, focused on the BOT side. B) Magnified regions of (A). as highlighted by the dashed orange boxes. ICAM‐1 expression co‐localized with the RFP‐HUVECs for TNF‐α‐treated chips, which is, to a lesser extent, the case for the control chips.

In addition to studying the inflammatory secretome in the SoC, we studied the expression of ICAM‐1 by the endothelial cells in the BOT compartment under inflammatory conditions, given its important role in monocyte adhesion, which is directly involved in leukocyte infiltration in arthritis [3, 9]. ICAM‐1 immunofluorescence staining revealed that the TNF‐α‐treated models displayed a strikingly more pronounced expression pattern (Figure 7A, columns 2 and 4), compared to the untreated controls (Figure 7A, columns 1 and 3). Indeed, zooming in on the cells (Figure 7B) revealed that the ICAM‐1 expression pattern overlapped with the RFP signal of the HUVECs in the TNF‐α‐treated models, while only a few cells presented such a pattern in the untreated control models. Of note, we observed diffuse expression of ICAM‐1 in untreated models (Figure 7A,B, columns 1 and 3), which is seemingly in contrast with previous work [29], in which little to no expression of ICAM‐1 was observed under untreated conditions. A possible explanation for this discrepancy is that, in our work, we employed FLS isolated from an OA patient, while in previous work [29], human subjects without a history of arthritis (healthy FLS) were considered. OA‐FLS are known to express ICAM‐1, especially after they have been expanded in vitro [15], as is the case in this work. ICAM‐1 expression by FLS in inflammatory conditions was next confirmed by confocal microscopy in the TOP compartment of a SoC device (Figure S6A). Therefore, it can still be concluded that TNF‐α stimulation of the SoC model, from the TOP compartment, increased ICAM‐1 expression by the endothelial cells in the BOT compartment. Combined with the Luminex assay outcomes, these data confirm the successful generation of an inflammatory phenotype in the SoC model.

## 3 Discussion

Cell‐cell interactions in the synovium, cell migration, and the release of inflammatory mediators are important events in the pathogenesis of arthritis [5, 7, 8, 13, 14, 20]. However, current in vitro synovium models do not always include the essential immune element, and only some of them support cell‐cell interactions at a relevant spatial scale. To address these limitations, we bioengineered and validated an alternative two‐compartment SoC platform comprising a resident immune component and patient‐derived FLS separated from a remodeling endothelial lumen by a 2‐µm‐thin microporous PDMS membrane that enables cell migration and multicellular interactions. Altogether, we propose a SoC platform that can be fabricated in a reproducible manner following a wafer‐scale manufacturing process (Figure S1) and which will be instrumental in addressing complex biological questions in the future in joint biology and joint‐related disorders.

With the main goal of mimicking the lining and sublining architecture of the synovium (Figure 1A) [1, 3], we have successfully established and maintained a triple co‐culture of patient‐derived FLS, HUVECs, and THP‐1‐derived macrophages for up to ten days in our devices, with expression of cadherin‐11, VE‐cadherin, and CD163. Confocal microscopy confirmed the recreation of an in vivo‐like synovium architecture, with THP‐1‐derived macrophages and FLS introduced on the top side of the membrane and HUVECs on the other side. Reproducible lumen formation through remodeling of the endothelium in the bottom compartment was observed from Day 5 onward in the SoC model, which was found to be associated with the migration of FLS from the TOP to the BOT compartment, to eventually surround the endothelium. These processes recreated a sublining‐like structure in the SoC, albeit with a single vascular lumen. In the future, it would be interesting to introduce lymphocytes and other immune cell types found in vivo to increase the physiological relevance of our model. In addition, endothelial sprouting from the BOT to the TOP compartment was observed, with striking changes in the endothelial cell morphology in the triple co‐culture models. Specifically, endothelial cells significantly elongated over time and aligned, even in the absence of fluid‐induced shear stress. These observations were consistent in all experimental runs, indicating that these changes were characteristic of the triple co‐culture model. For future work, it would be interesting to study whether those changes are inherent to the single FLS donor we have considered in these experiments or if they could be extended to multiple donors, ideally derived from different arthritic disease conditions (OA, RA). Interestingly, in previous work, also using a similar two‐compartment SoC [29] and employing healthy FLS, neither changes in endothelial morphology nor lumen remodeling were reported, suggesting that the exact phenotype of the FLS, of either OA or of healthy origin, might impact the FLS migratory potential and effect on the endothelium. To test this hypothesis, additional experiments using multiple donors from both healthy and OA origins will be conducted in future work, since they fall beyond the scope of the present paper. Alternatively, these discrepancies might be explained by the dimension differences in the porous membrane (50 *vs*. 2 µm thickness) or the employed coating strategy that could alter the FLS migratory ability. Given the absence of any physiological membrane separating the lining and sublining layers in vivo, employing the thinnest membrane possible is desirable. Therefore, the thin membrane used in our SoC platform may be advantageous to study synovial tissue remodeling and, in turn, to increase our understanding of multicellular interactions in the synovium.

In recent years, increasingly more FLS phenotypes have been discovered in OA and RA [4, 15, 16, 41], and it is well known that studying the FLS in isolation can introduce a bias since a cell's phenotype is highly dependent on its microenvironment [40, 41]. Therefore, our SoC platform could be used to screen different FLS phenotypes in a more relevant environment. Furthermore, we hypothesize that by incorporating various arthritis‐related FLS phenotypes, a disease model can be created not only to eventually aid in increasing our understanding of arthritis, but also in targeted drug development. For example, by relating the FLS phenotypes to OA patient stratification, our SoC model could aid in developing patient group‐specific therapeutics [49].

For this work, mainly aiming at developing a technological platform for studying arthritis and mechanistic biology in future studies, we employed THP‐1‐derived macrophages in our SoC model as a resident immune component. This cell line should ultimately be replaced by more mature and disease‐relevant macrophage phenotypes generated from primary monocytes isolated from patient blood [33, 50], and ideally from the same patient as the FLS, in the view of creating patient‐specific models. It should be noted that the same SoC protocols reported here can be a priori followed for primary monocyte‐derived macrophages. Yet, some optimization may be needed, particularly for the cell detachment method to preserve surface proteins, as some macrophage subsets could be more challenging to detach from their substrates enzymatically.

Recently, new macrophage subsets have been identified for both RA and OA. For RA, it has been shown that non‐resident monocyte‐derived macrophages that infiltrate from the bloodstream are crucial in the progression of the disease [18]. Similarly, a subset of CD163^+^CD206^+^ macrophages, classically viewed as M2 macrophages, was found to be pro‐inflammatory with enhanced CD40 expression in RA compared to healthy controls [51]. In OA, macrophages display a mixed phenotype that can be recreated in vitro by stimulation with inflammation‐related proteins from the S100 family (S100A8/A9) [19]. Furthermore, co‐culturing pathogenic macrophage subsets, or the use of conditioned medium, has been shown to induce inflammatory phenotypes in otherwise healthy FLS [18, 51]. In future work, we intend to explore the applicability of our SoC platform to study the effects of various macrophage subsets on the model and the disease progression. In addition, the resident immune component, when using both a macrophage source and phenotype suitable for studying a particular disease state, provides the unique opportunity to study immune‐targeting therapies [52, 53] in a complex and relevant in vitro environment, which is crucial as previous SoC work has already shown that resident macrophages change their functionality and transcriptomic profile in co‐cultures with FLS [33].

A limitation of our current SoC model is the absence of mechanical stimulation [21], which is common during joint locomotion in vivo and has been shown to be crucial for, for example, cartilage differentiation in vitro [54, 55]. Some in vitro studies have hinted at the effects of mechanical stimulation on the synovial membrane in OA as well [29, 56]. Our platform could be adapted to enable these studies with the incorporation of pneumatic actuation chambers, which, combined with the 2 µm‐thin elastomeric porous membrane, would allow for dynamic stretching of the cells cultured on the membrane, inspired by previous work [57].

Another limitation is the unknown and potentially heterogeneous role of the THP‐1‐derived macrophages in our triple co‐culture model, which should be studied more in‐depth in the future to uncover FLS‐macrophage interactions, which are expected to be relevant in arthritis. In this study, which serves as a proof‐of‐concept for the proposed SoC platform, the functional state of the macrophages was not studied in detail, given the potentially heterogeneous response of THP‐1 to polarizing stimuli on the surface marker level [50, 58]. Using more mature macrophages derived from PBMCs, as mentioned previously, future studies could unlock the potential of our platform to investigate macrophage‐FLS‐endothelium interactions in a controlled environment.

Finally, a single OA‐FLS donor was considered in this work, in view of establishing and validating the platform and the triple co‐culture. Given that the OA‐FLS cells were derived from a diseased tissue, inflammatory or fibrotic processes may have already been triggered in our control experiments, in the absence of TNF‐α stimulation. Still, a significant difference in response to the TNF‐α stimulation could be observed, with the upregulation of IL‐6, IL‐8, CCL2, and GM‐CSF, illustrating that our model supports relative comparisons to unstimulated controls and basic inflammation studies.

## 4 Conclusion

In this work, we established a SoC platform and positioned it as an enabling technology for studying the synovial membrane in vitro. Our SoC platform comprises a resident macrophage component, patient‐derived FLS, and an endothelial lumen in a device that includes a thin microporous membrane supporting direct and indirect cell‐cell communication and cell migration. We have revealed that interactions between FLS and endothelial cells lead to cell migration and on‐chip tissue remodeling, thereby mimicking key aspects of synovial membrane biology in healthy and arthritic disease conditions. We have demonstrated that our SoC model responds to an induced inflammatory response in the context of arthritis. Altogether, we believe that our SoC platform will be instrumental in studying the role of the synovial membrane in joint health and disease, with potential for application in drug development, through new target discovery and drug screening, possibly in a patient‐specific manner.

## 5 Experimental Section

### 5.1 Chip Design & Fabrication

The SoC devices consisted of four layers: the bottom and top fluidic layers, each comprising one channel (BOT and TOP channel, respectively), a reservoir layer, and a thin microporous membrane between the top and bottom layers. Wafer‐scale molds (20 chips per mold, Figure S1A) for the reservoir, top, and bottom layers were designed in SolidWorks (2023 SP5.0, Dassault Systèmes, Waltham, MA, USA) and fabricated from 5‐mm thick PMMA sheets using CNC micro‐milling (Neo, Datron A.G., Mühltal, Germany) after designing milling operations using Fusion software (v2.0.20981, Autodesk, San Francisco, CA, USA). Next, the PDMS curing agent and base polymer (Sylgard 184, Dow Chemical, Midland, MI, USA) were mixed in a 1:10 wt.% ratio, degassed thoroughly, cast on the PMMA molds, and cured overnight at 60°C.

The thin microporous membranes (approximately 2 µm thick, 5‐µm‐diameter pores, 30‐µm pitch) were fabricated out of PDMS as reported in previous work from our group (Figure S7) [34, 35]. Briefly, 4″ silicon wafers (Okmetic, Vantaa, Finland) were cleaned with HNO_3_, primed with hexamethyldisilazane (HMDS), and spin‐coated with a 10 µm layer of AZ10XT 520cp positive resist (MicroChemicals GmbH, Ulm, Germany). A quartz‐chromium photomask to pattern 5 µm pillars was designed using CleWin (v5.4, WieWeb software, Hengelo, the Netherlands) and fabricated in the MESA+ NanoLab (University of Twente, Enschede, the Netherlands). The pillars were patterned using the photomask and subsequently developed in OPD 4262 (Fujifilm Netherlands BV, Lochem, the Netherlands). A PDMS mixture (same composition as above) was diluted 2:5 (wt.%) in *n*‐Hexane (Sigma–Aldrich, Zwijndrecht, the Netherlands) and spin‐coated to form a thin layer covering the entire wafer surface and the pillars. After curing at 60°C overnight, the PDMS‐covered wafers were etched with a custom reactive ion etching system (TEtske, MESA+ Nanolab) using a mixture of SF_6_ and O_2_ gases to remove the thin layer covering the pillars, thereby creating a porous membrane.

The final SoC chips were assembled at the wafer scale by sequentially bonding the various layers using oxygen plasma treatment (Femto Science CUTE, Hwaseong‐si, Gyeonggi‐do, Republic of Korea) (Figure S1B). First, the reservoir and top layers were aligned manually and bonded together, followed by a post‐bonding bake at 60°C for 1 h. For the membrane transfer, this assembly was then bonded to the wafer supporting the etched membranes, followed by another bake at 60°C for at least 1 h. The membrane was lifted from the wafer by placing the assembly in technical‐grade acetone for 7 min with mild manual agitation to dissolve the AZ10XT photoresist. Next, the resulting assembly was washed in technical‐grade isopropanol (IPA) and dried overnight in a fume hood, after which the bottom layer and the resulting assembly were aligned manually, bonded, and baked at 60°C for at least 1 h. Finally, the membrane's integrity was verified by microscopic inspection, and chips were cut using a scalpel. Before device use, both channels were washed once with acetone to remove residual photoresist and three times with 70% ethanol for sterilization.

### 5.2 Cell Culture

Human knee FLS were obtained with written informed consent from a female patient (61 years old, end‐stage OA) undergoing total knee replacement surgery according to the Maastricht UMC Medical Ethical regulations and were kindly provided by Prof. dr. Tim Welting (Maastricht UMC, Maastricht, the Netherlands). FLS were cultured in Dulbecco's Modified Eagle's Medium/Ham's F12 nutrient mixture with GlutaMAX (DMEM/F12‐GlutaMAX, Gibco, Thermo Fisher Scientific, Waltham, MA, USA) supplemented with 10% Fetal Bovine Serum (FBS, Sigma–Aldrich), 1% Penicillin/Streptomycin (Pen/Strep, Gibco), and 1% Modified Eagle's Medium Non‐essential amino acids (MEM‐NEAA, Gibco) (FLS medium) in collagen‐1 coated (Gibco) tissue‐culture treated T175 (Greiner Bio‐One, Frickenhausen, Germany) and T300 (VWR, Radnor, PA, USA) culture flasks in a humified incubator at 37°C and 5% CO_2_. Full medium replacement was performed three times a week. FLS were passaged using a 0.25% Trypsin‐EDTA solution (Gibco) upon reaching 80–85% confluence and were used at passages 6–8.

Red Fluorescent Protein expressing Human Umbilical Vein Endothelial Cells (RFP‐HUVECs, cat. no. CAP001‐RFP, Angio‐Proteomie, Boston, MA, USA) were cultured in Endothelial Growth Medium 2 (EGM2, Promocell GmbH, Heidelberg, Germany) supplemented with 1% Pen/Strep on collagen‐1 pre‐coated T75 culture flasks (Greiner Bio‐One CellCoat collagen 1) in a humidified incubator at 37°C and 5% CO_2_. RFP‐HUVECs were passaged using a 0.25% Trypsin‐EDTA solution upon reaching 80–85% confluence and were used at passages 5–6.

THP‐1 monocytes [36] (ATCC no. TIB‐202, cat. no. C0003024, CliniSciences Cell Lines Service, Amsterdam, the Netherlands) were cultured in suspension in Roswell Park Memorial Institute 1640 (RPMI‐1640, Gibco) medium, supplemented with 10% FBS and 1% Pen/Strep at an initial density of 1·10^5^–2·10^5^ cells mL^−1^ in upright T25 or T75 culture flasks (Greiner Bio‐One). THP‐1 cells were passaged twice a week. Macrophage differentiation was induced by stimulating the cells at a density of 2·10^5^ cells mL^−1^ with 50 ng mL^−1^ Phorbol‐12‐myristate‐13‐acetate (PMA, Sigma–Aldrich) in DMSO for 48 h, followed by 24 h of rest in PMA‐free culture medium [38, 59]. Stimulated THP‐1 adhered to the culture flask, exhibited a round‐to‐stellate morphology with granular cytoplasm, and expressed cluster of differentiation 68 (CD68, pan‐macrophage marker) (Figure S8). Differentiated THP‐1 cells were detached before experiments using Accutase (Stemcell Technologies, Vancouver, BC, Canada) for 15 min to preserve their viability and surface proteins, as previously described [60, 61].

### 5.3 Establishment of a Triple Co‐Culture in the SoC

The experimental timeline for all SoC experiments reported in this paper is presented in Figure 2A. On Day ‐1, plasma‐activated SoC devices were incubated with a 2 mg mL^−1^ polydopamine solution in Tris‐HCl at pH 8.5 (dopamine‐HCl, Sigma–Aldrich) for 30 min at room temperature, washed three times with sterile PBS, and finally coated with 100 µg mL^−1^ rat‐tail collagen 1 (Gibco, lot 963787) for 1 h at 37°C in a humidified incubator. The channels were washed twice with PBS and flushed with FLS medium before use. Next, 25 µL of an FLS cell suspension (2·10^6^ viable cells mL^−1^), either labeled or not with CellTracker green CMFDA (Invitrogen, Thermo Fisher Scientific) according to the manufacturer's instructions, was injected into the top channel, and FLS were allowed to adhere overnight.

On the next day (Day 0), RFP‐HUVECs were seeded in the bottom channel using a two‐step approach (Figure S9). 20 µL of an RFP‐HUVEC cell suspension (4.5·10^6^–6·10^6^ cells mL^−1^) was loaded in the bottom channel, followed by at least a 1.5 h incubation to promote cell attachment. Next, the outlets of the bottom channel were emptied, and another 20 µL of freshly prepared cell suspension (4.5·10^6^–6.0·10^6^ viable cells mL^−1^) was injected into the bottom channel, after which the chips were immediately inverted to promote cell attachment to the bottom side of the membrane. After at least 1.5 h of incubation, the chips were flipped back, and EGM2 was flushed through the bottom channel to remove cells not adhering to any surface. Finally, the medium in the top channel was refreshed with FLS medium, and the SoC devices were cultured overnight in a humidified incubator.

On Days 1 and 2 (Figure 2A), the medium of each channel was refreshed with FLS medium (TOP) or EGM2 (BOT), and monolayer formation of FLS and RFP‐HUVECs was assessed using an EVOS inverted fluorescence microscope (Thermo Fisher Scientific). SoC devices with noticeable gaps in either of the monolayers on Day 2 were excluded from the study.

On Day 3 (Figure 2A), THP‐1 macrophages were labeled with CellTracker green CMFDA (Invitrogen) according to the manufacturer's instructions if required and seeded in the top channel at a concentration of 8.5·10^5^–9.0·10^5^ viable cells mL^−1^. Macrophages were allowed to attach for approximately 42 h (Day 3 + Day 4) before the medium was refreshed on Day 5. Starting on Day 3, THP‐1 medium was employed in the top chamber instead of FLS medium to preserve the macrophages.

Finally, from Day 5 to Day 10 (Figure 2A), SoC devices were observed daily using an EVOS microscope. Each day, the media in the top and bottom channels were replaced with THP‐1 and EGM2 media, respectively.

### 5.4 Quantification of Endothelial Lumen Projected Width

Endothelial lumen projected diameters were quantified on Days 2, 3, and 5–10 (Figure 2A) using a custom MATLAB (R2022b, The MathWorks Inc., Natick, MA, USA) script (available upon request). As input for this script, low‐magnification images were taken for each SoC device, covering about 2/3 of the channel length, using both the brightfield and RFP channels, using an EVOS microscope. First, the leftmost channel wall on the brightfield image was manually selected to determine the orientation of the chip. Next, in the corresponding RFP image, intensity profiles were measured along the width of the channel (edge regions were excluded since the lumen edges were not entirely in the field of view), resulting in an average of 75 profiles per image (25 µm spacing). The lumen edges on each profile were detected using an Otsu threshold multiplied by an empirical correction factor. The projected width was calculated based on the Euclidean distance between the edges detected on each profile (Figure S3). The average projected width for each chip was calculated and reported here. Statistical analysis was performed using a linear mixed model (LMM) analysis as described below.

### 5.5 FITC‐Dextran Endothelial Lumen Perfusability Assay

To assess the perfusability of the endothelial lumen formed in the SoC devices, we injected 4 kDa FITC‐Dextran in the bottom compartment of the SoC devices at Day 10. Specifically, a stock solution of 1 mg mL^−1^ 4 kDa FITC‐Dextran (Sigma–Aldrich) was prepared in sterile PBS, which was then further diluted to a final concentration of 100 µg mL^−1^ in EGM‐2 medium. The reservoirs of the bottom compartment were emptied, and 40 µL of this FITC‐Dextran solution was injected, while the chip was already placed on the microscope stage (Leica DMI5000M, Leica Microsystems GmbH, Wetzlar, Germany). Next, the chips were imaged, starting 1 min after injection, at 2‐min intervals using a Leica HC PL FLUOTAR 10x/0.30 objective, a CoolLED PE‐300ultra 450 nm light source (CoolLED ltd., Andover, United Kingdom), a quad filter with suitable bandpass filters for the detection of FITC (excitation filter: 478/33 nm, emission filter: 518/26 nm; Chroma 89402ET, Chroma Technology GmbH, Olching, Germany), and a FLIR monochrome CMOS camera (GS3‐U3‐23S6M‐C, Teledyne FLIR, Täby, Sweden). Images were processed in Fiji for linear contrast enhancement.

### 5.6 Immunofluorescence & Confocal Microscopy

For immunofluorescence and confocal microscopy SoC chips were washed twice with regular PBS (containing Mg^2+^ and Ca^2+^) and subsequently fixed using 4% paraformaldehyde for 20 min at room temperature, followed by three washing steps with PBS. Samples were stored at 4°C until analysis.

For immunofluorescence staining of cadherin‐11, CD90/THY1, CD163, VE‐cadherin, and CD68, fixed samples were washed with PBS, permeabilized using a 0.25% vol Triton‐X100 solution (Sigma–Aldrich) in PBS for 10 min at room temperature, and subsequently blocked using 5 wt.% bovine serum albumin (BSA, Sigma–Aldrich) in PBS Tween 20 (0.1% vol PBST, Sigma–Aldrich) or a mixture of normal goat serum (8% vol, Invitrogen) and BSA (2 wt.%) for 1 h at room temperature (Table S1). Primary antibodies (Table S1) were diluted in a 2% BSA solution in PBST and incubated overnight at 4°C. Samples were then washed three times for 5 min with 0.5% PBST, followed by incubation with appropriate Alexa Fluor 488‐ or 647‐conjugated secondary antibodies (Invitrogen) and subsequent washes (3 or more) with 0.5% PBST. Nuclei were counterstained with NucBlue (Invitrogen ReadyProbes) or 4',6‐diamidino‐2‐phenylindole (DAPI, Thermo Fisher Scientific), and if applicable, actin filaments were stained using ActinGreen 488 (Invitrogen ReadyProbes).

For ICAM‐1 immunofluorescence staining, the primary antibody incubation was performed on unfixed samples (see antibody test result for TNF‐α‐treated HUVECs in Figure S6B). Briefly, the ICAM‐1 primary antibody (Table S1) was diluted in EGM2, injected in the bottom channel of the SoC chips, and incubated for 4 h in a humidified incubator at 37°C and 5% CO_2_. Afterward, the chips were washed three times with regular PBS and fixed, and the samples were further stained as described above.

Next, all the stained chips were imaged in the University of Twente BioImaging Centre (BIC, Enschede, the Netherlands) facility using a Zeiss LSM880 laser scanning confocal microscope controlled by Zen Black software (both Carl Zeiss Microscopy GmbH, Jena, Germany). Samples were imaged without chip disassembly, except when higher spatial resolution in the z‐direction was required (e.g., for resolving the cell layers on either side of the membrane). In that case, devices were carefully disassembled using a surgical blade and imaged with a higher numerical aperture objective (20×0.8 or 63×1.2 W objectives, Zeiss). Images were processed further using Zen Blue Lite (Zeiss) or a combination of Fiji [62] and machine learning in Python for morphological characterization, as discussed below.

### 5.7 Label‐Free Confocal Reflection Microscopy

Confocal reflection microscopy was employed to visualize the cell layers in the SoC devices and the unlabeled porous PDMS membrane simultaneously. To this end, fixed SoC devices were disassembled, and the TOP compartment with the membrane was placed on a #1.5 coverslip (Menzel Gläser, VWR). Next, confocal reflection imaging was performed using a Zeiss LSM880 confocal microscope with the appropriate transmission/reflection mirror block, the 488 nm Argon laser, and a Zeiss C‐Apochromat 63 × 1.2 W Autocorr objective with the corresponding immersion fluid.

### 5.8 Morphological Analysis of the VE‐Cadherin‐Stained Endothelium Using Cellpose

To quantify the morphological change of the HUVECs, VE‐cadherin confocal microscopy images were analyzed using machine‐learning‐assisted segmentation. Our approach (Figure S10A) was based on a protocol recently published by Jiao et al. [63]. First, all VE‐cadherin images were preprocessed using batch processing in Fiji to remove shot noise and enhance contrast. Next, we used Cellpose [42, 43, 44] to segment cells based on their VE‐cadherin signal. We trained a custom Cellpose model using “human‐in‐the‐loop” training [43] on a subset of the data (four confocal images, 220–410 regions‐of‐interest (ROIs), Figure S10B) that represented the various cell morphologies found in our complete data set. The basic Cellpose3 model with denoising [44] was iteratively specialized to our dataset by training it first on a cropped subset of the training dataset (Figure S10C), to reduce the number of manual annotations required. The resulting model was then further specialized using the full‐size images (Figure S10B). This final custom Cellpose model was used to analyze all images of all time points, and all outlines, and masks were saved for verification and further processing. Then, the masks generated by Cellpose were processed in bulk using MorphoLibJ [64] to convert them to ROIs inside Fiji and to remove all cells on the borders of the image. Finally, the LabelsToROIs plugin [65] was used to calculate cell shape descriptors from the ROIs. Before calculations were performed, the segmentation ROIs were verified manually, and faulty segmentations (over‐ or under‐segmentation, non‐cell ROIs) were removed manually before analysis. The updated ROIs were saved for future reference. Figure S10D depicts an example output of the segmentation algorithm. All shape descriptors from LabelsToROIs were imported into MATLAB, and ROIs smaller than 200 µm^2^ were filtered out, as these typically correspond to small gaps between cells or are artificial ROIs resulting from optimizing the segmentation model to work on elongated cells, which are known to be more challenging to segment [42]. Statistical analysis was performed as described below, and the full test output can be found in Tables S2–S4.

### 5.9 Generation of an Inflammatory Phenotype and Luminex Secretome Analysis

To generate an inflammatory phenotype in the SoC devices, the FLS and macrophages in the TOP channel were stimulated with 10 ng mL^−1^ recombinant human TNF‐α (BioLegend, San Diego, CA, USA) diluted in THP‐1 medium. TNF‐α was added daily from Day 6 to Day 10 to the TOP channel (Figure 6A), and 10 µL supernatant medium samples were collected from Day 7 to Day 10 from the same channel and stored at −80°C until further analysis.

A Luminex assay (Bio‐Rad, Hercules, CA, USA) was performed following the manufacturer's instructions to quantify the secretion of IL‐6, IL‐8, IL‐10, IL‐1β, granulocyte‐macrophage colony‐stimulating factor (GM‐CSF), and CC chemokine ligand 2 (CCL2) in the top channel of the SoC. Samples were prepared by pooling all supernatant media aliquots (10 µL) from the same condition for each experimental run at each time point (4‐10 chips per time point). Each data point represents one of the three independent experimental runs performed in this study.

### 5.10 Statistical Analysis

Unless otherwise noted, measurement data were not transformed, and statistical analysis was performed in OriginPro (v2023, OriginLab, Northhampton, MA, USA) for multiple‐comparison testing or in SPSS Statistics (v28.0.1.0, IBM, Armonk, NY, USA) for linear mixed model analysis. The number of samples and the meanings of the statistical significance symbols are given in the Figure captions.

Statistical significance for differences in the trends of the average projected lumen for the HUVEC only and the triple co‐culture conditions over time was tested using a linear mixed model (LMM) analysis with unstructured covariance (effects: time point and the group (HUVEC only/triple co‐culture) and their interaction (time*group), no random effects) in SPSS. The LMM prediction was plotted with 95% confidence intervals, and significance was determined by the time point for which these intervals did not overlap between the control (HUVEC only) and triple co‐culture conditions.

Statistical analysis of endothelial morphology based on the Cellpose output was performed in OriginPro. Data for multiple SoC chips per condition were pooled, and Statistical significances were tested by a Kruskal‐Wallis ANOVA with Dunn's post‐hoc test.

Statistical analysis for the Luminex assay of secreted inflammatory factors was not performed since a normal distribution could not be assumed, given the low sample number. This would require rank‐based testing, which is not valid for N = 3 per condition.

## Author Contributions

Conceptualization was carried out by L.R.S., N.A.G., M.H.J.V.D.B., L.I.S., S.L.G., and M.K. Methodology was developed by L.R.S., F.R.S., N.A.G., M.A.H., and M.H.J.V.D.B. Investigation was performed by L.R.S., F.R.S., N.A.G., D.W., M.A.H., and M.H.J.V.D.B. Visualization was conducted by L.R.S. Supervision was provided by L.I.S., S.L.G., and M.K. Writing – original draft was prepared by L.R.S., and writing – review and editing were undertaken by L.R.S., F.R.S., N.A.G., L.I.S., S.L.G., and M.K.

## Funding

This work was supported by the Twente Graduate School Award 2022 issued by the University of Twente to LRS and by the TopTreat grant from the Holland High Tech, topsector High Tech Systems and Materials with a PPS innovation subsidy for public‐private partnerships in research and development to LRS, FRS, and MK, and by ReumaNederland with grant number LLP25 to MK. This publication is part of the Organ‐on‐Chip Centre Twente project “Organ‐on‐Chip Development Center (hDMT INFRA OoCDev)” with file number 175.2021.005 of the research programme Research Infrastructure: National Consortia which is (partly) financed by the Dutch Research Council (NWO).

## Conflicts of Interest

The authors declare no conflicts of interest.

## Supporting information




**Supporting File**: adhm70757‐sup‐0001‐SuppMat.pdf.

## Data Availability

All data needed to evaluate the observations and conclusions in this paper are available in the main text and/or the supplementary materials. MATLAB codes for the analysis of projected widths of endothelial lumens and the Cellpose models used are available upon request. Similarly, the OoC models will be made available in collaboration upon reasonable request for other tissue models or similar Synovium‐on‐Chip studies.
