# Supplementary material for: A Synovium‐on‐Chip Platform to Study Multicellular Interactions in Arthritis
Source: Adv Healthc Mater. 2026 Jan 24;15(15):e04857. doi: 10.1002/adhm.202504857 (PMC13088745; doi:10.1002/adhm.202504857)
Supplement: Supplementary file 1 — Supporting File: adhm70757‐sup‐0001‐SuppMat.pdf. [file ADHM-15-0-s001.pdf]

SUPPLEMENTARY INFORMATION
A Synovium-On-Chip Platform to Study Multicellular Interactions in Arthritis

Laurens R. Spoelstra *et al.*

Corresponding authors: l.i.segerink@utwente.nl (porous membrane technology) s.legac@utwente.nl (organ-on-chip design, vasculature), marcel.karperien@utwente.nl (arthritis and cell biology)

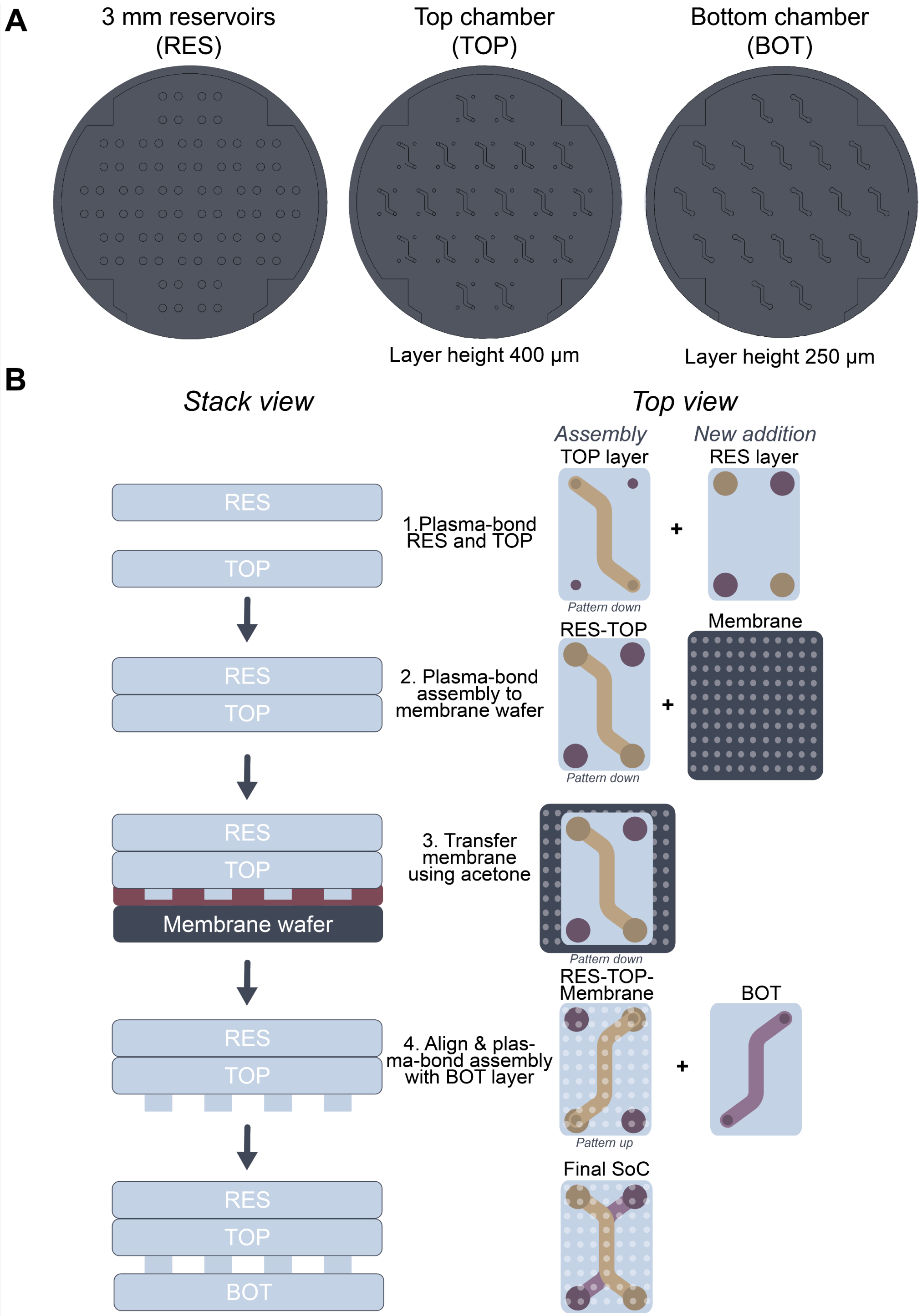

Figure S1. Synovium-on-chip (SoC) fabrication procedure. A) Schematic images of 20-chip computer numerical control (CNC)-milled PMMA molds used for wafer-scale chip fabrication. B) Schematic overview of the SoC assembly protocol. *Left:* layer representation (side view), *right:* top view of assembly steps. PDMS is shown in light blue, the two culture channels in yellow (TOP) and red (BOT), the silicon support wafer for the membrane fabrication in dark gray, the photoresist used to make the pores in dark red (see also Figure S6) and the membranes pores in light gray.

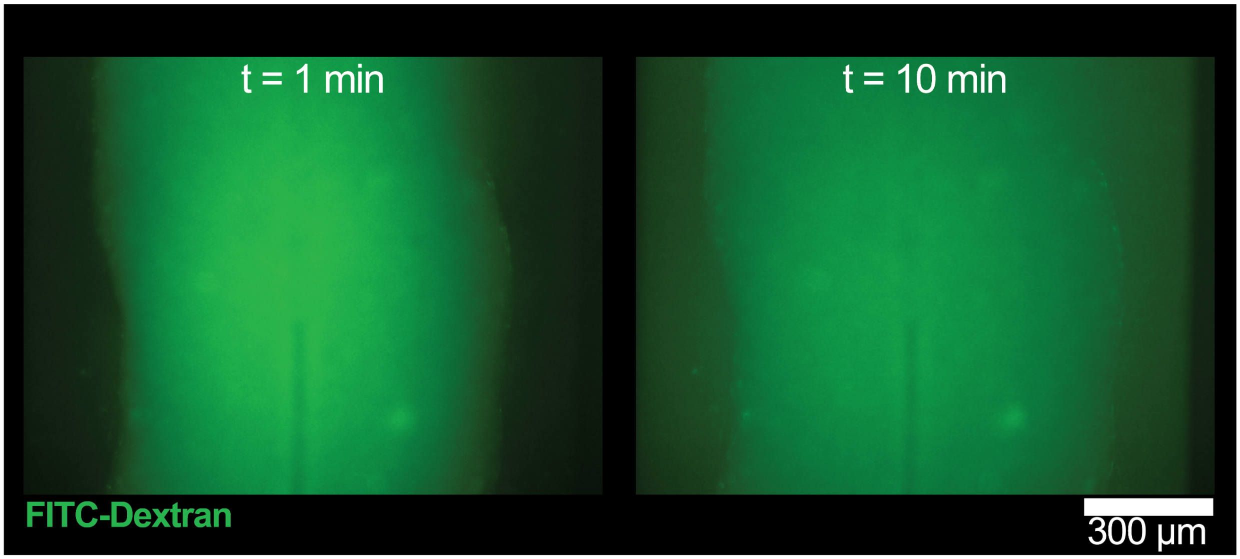

Figure S2. 4 kDa FITC-Dextran perfusion of endothelial lumens in the BOT compartment on Day 10. Initially, the lumen retains the small FITC-dextran molecules (*left*), but after 10 minutes (*right*) fluorescent signal was detected next to the lumen, indicating that the FITC-Dextran has diffused out of the lumen.

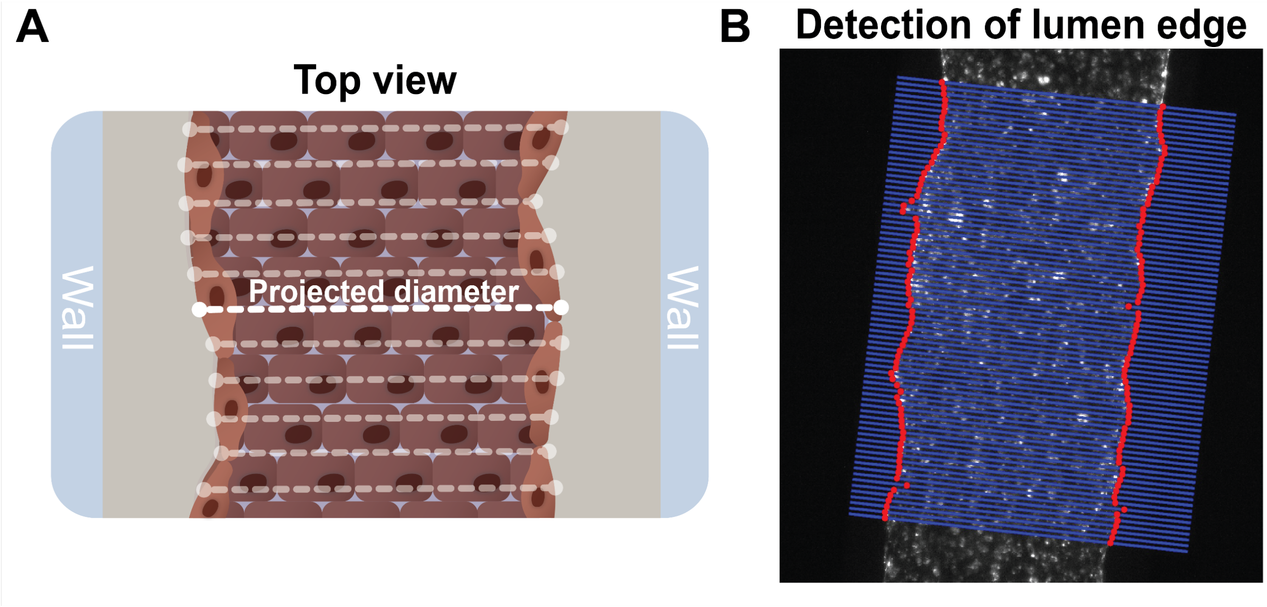

Figure S3. Determination of endothelial lumen projected with using MATLAB. A) Schematic representation of projected with definition. Dashed lines represent the individual profiles measured. **B)** Example output for a Day 10 endothelial lumen (RFP image). Blue lines represent the individual profiles measured in MATLAB. Red dots represent the detected lumen edge on the profile. The Euclidean distance between these points defines the projected width on the profile. The average for all profiles in an image is taken and compared between samples and over time.

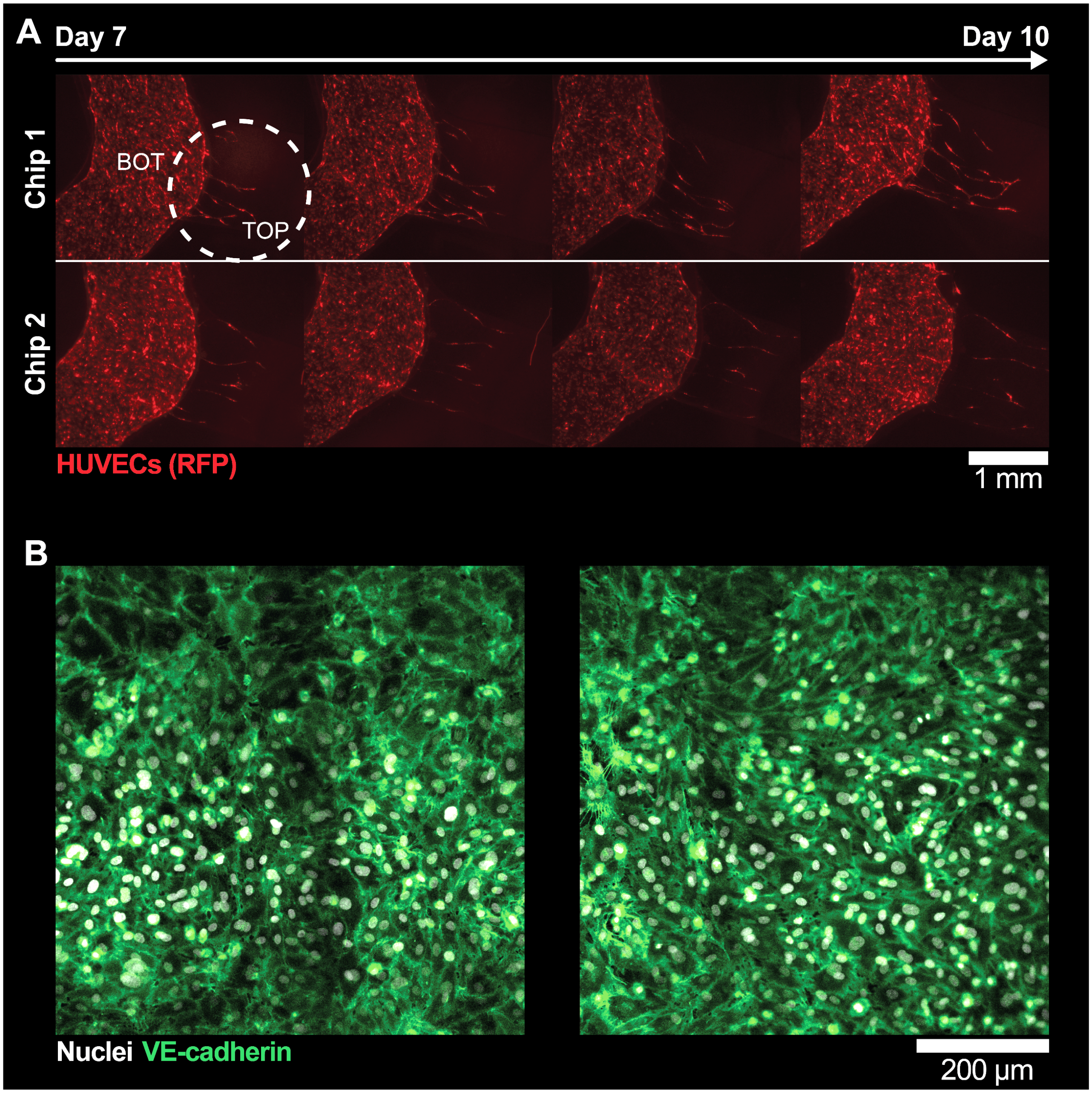

Figure S4. Angiogenic sprouting in triple co-culture samples and endothelial morphology in HUVEC-only samples (controls). A) Widefield fluorescence microscopy images for RFP-HUVEC lumens of two representative chips from Day 7 (*left*) to Day 10 (*right*, 1 image/day). Notice the endothelial sprouts in the bottom lower right corner (TOP compartment), originating from the BOT compartment (see white dashed area shown in top left image for reference). **B)** Confocal microscopy images of VE-cadherin expression in HUVEC-only controls (1 image/representative chip) on Day 10 (membrane-attached cells). Notice the cobblestone-like morphology of the HUVECs. Of note: HUVECs on their own displayed a more stressed and generally slightly unhealthier phenotype compared to triple co-culture models, most likely as a result of the absence of other cell types in the model.

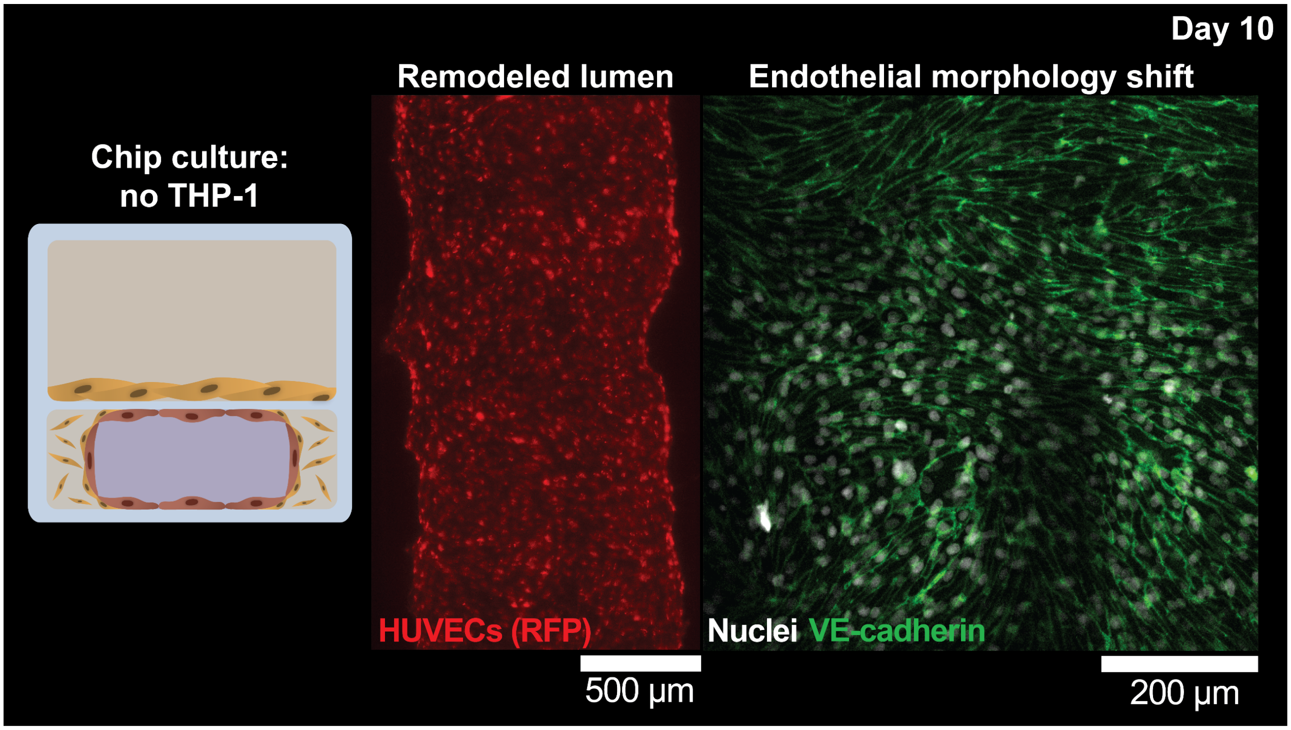

Figure S5. Endothelial lumen remodeling and phenotypic shift in FLS-HUVEC co-cultures in the SoC. *Left:* effective co-culture with FLS migrating through the membrane to surround the HUVECs in the BOT compartment. *Middle:* RFP-positive endothelial lumen on Day 10. *Right:* VE-cadherin immunofluorescence showing elongated endothelial cells, even in the absence of THP-1-derived macrophages.

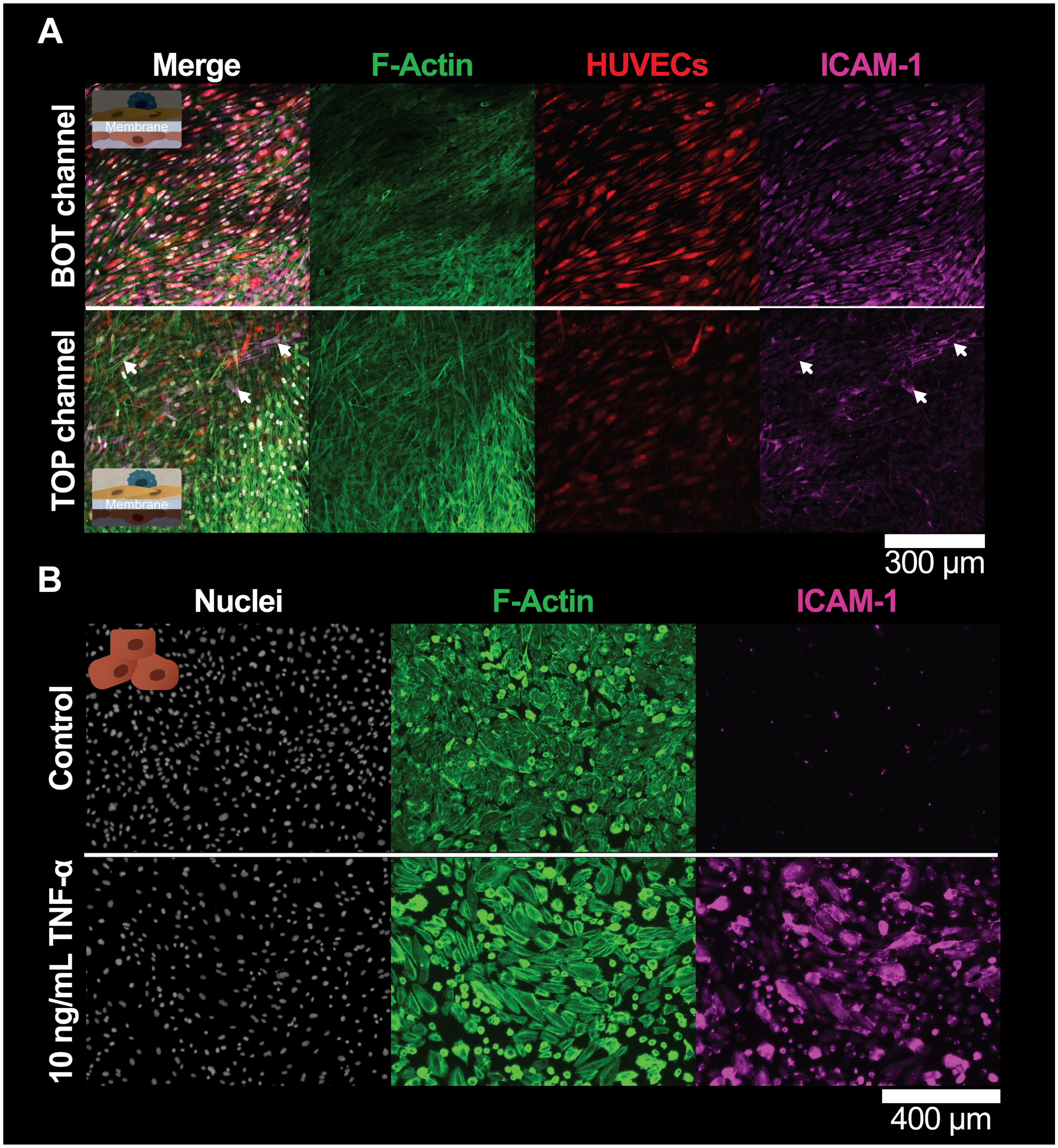

Figure S6. ICAM-1 immunofluorescence signal on FLS and HUVECs in the SoC devices and on HUVECs cultured on coverslips as a control. A) ICAM-1 expression patterns by HUVECs and FLS in a 10 ng/mL TNF-α-treated SoC device. Notice how the FLS in the TOP channel (RFP-negative cells) identified by the F-Actin staining pattern are positive for ICAM-1 (selected examples indicated with arrows). **B)** ICAM-1 antibody test with live-cell primary antibody incubation on HUVECs cultured on coverslips with and without 10 ng/mL TNF-α. Notice how the ICAM-1 signal is limited in control wells, but highly upregulated in the presence of TNF-α.

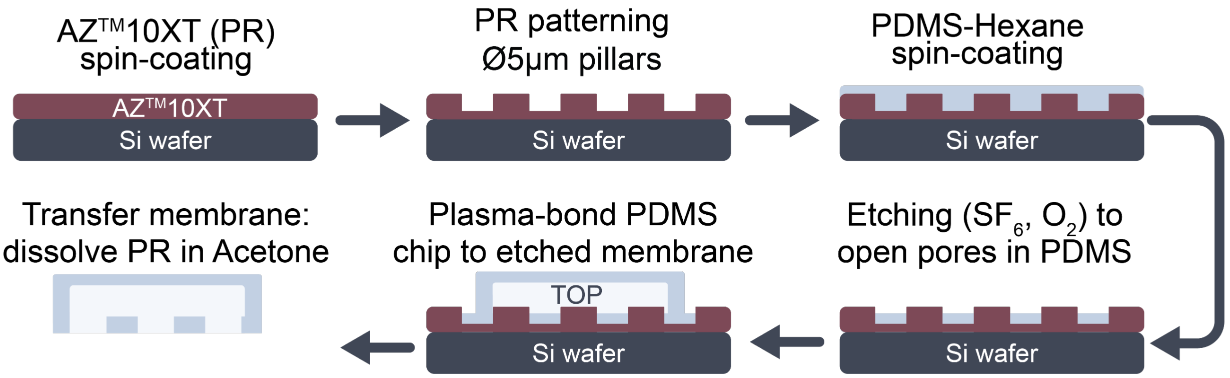

Figure S7. Schematic overview of porous PDMS membrane (5 µm pores, 30 µm spacing, ~2 µm thick) fabrication. Note that the final 2 steps (plasma bonding + transfer) are described in more detail in Figure S1B.

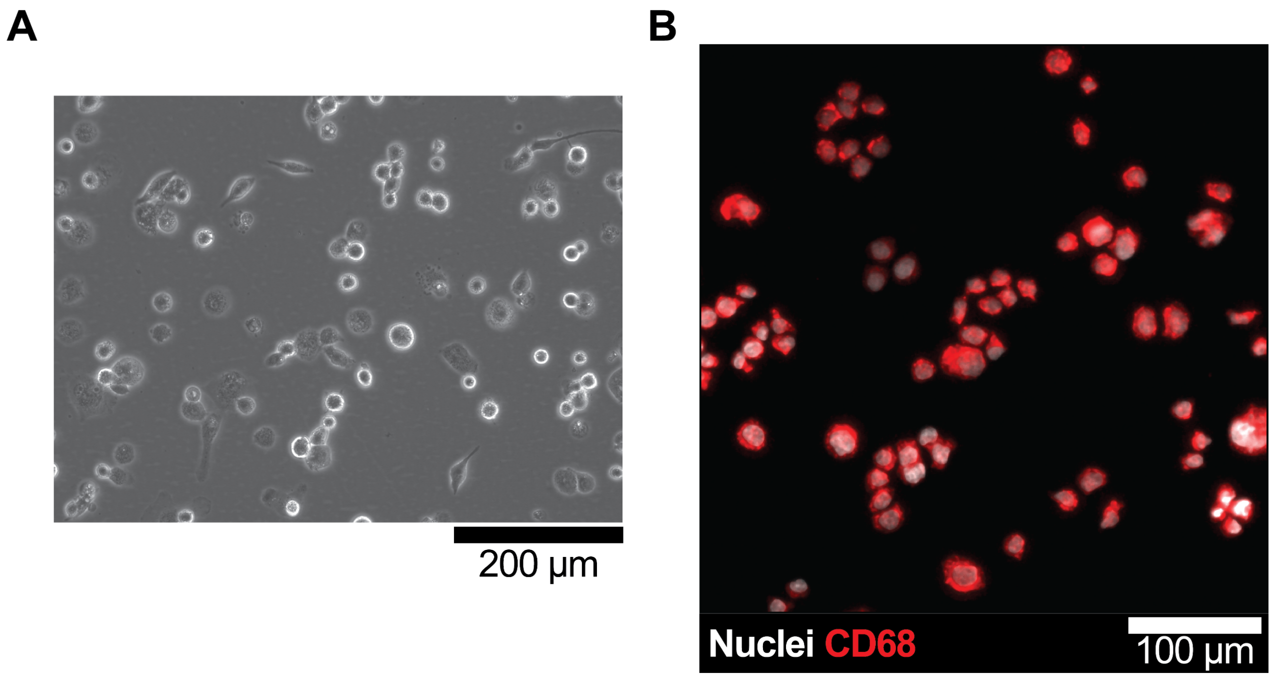

Figure S8. THP-1-derived macrophages after 48 h stimulation with 50 ng/mL of PMA and 24 h rest in PMA-free medium before harvesting for seeding in the SoC devices. A) Phase contrast microscopy image of THP-1-derived macrophages displaying round to stellate morphology with granular cytoplasm. **B)** Immunofluorescence image for CD68 expression (red) on THP-1-derived macrophages. Nuclei were counter-stained with DAPI (white).

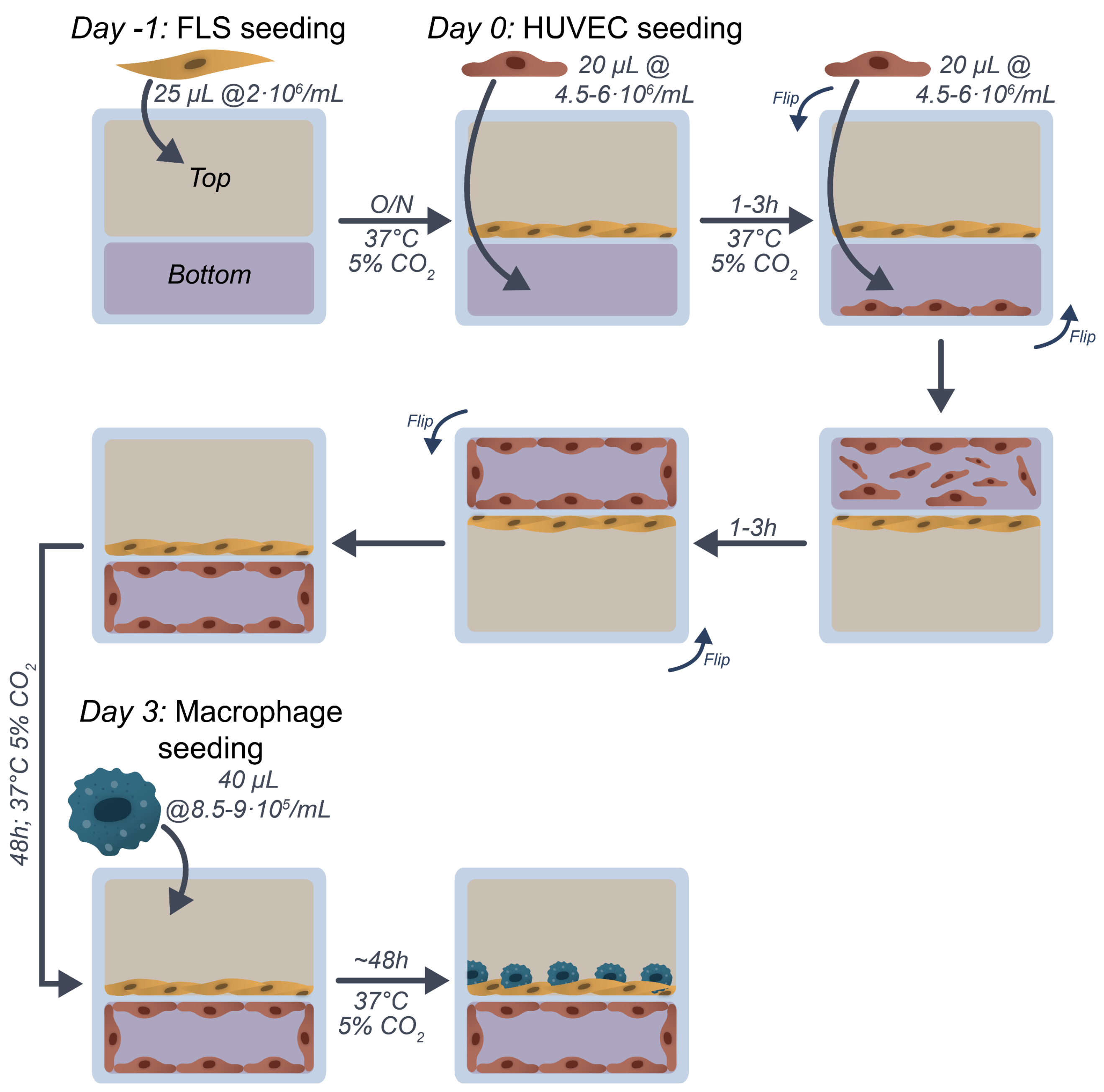

Figure S9. Schematic overview of the two-step seeding procedure at the start of the experiment to create an FLS lining in the top chamber and an endothelialized bottom chamber. Macrophages are seeded on Day 3 after 48 h incubation of the FLS and HUVEC co-culture.

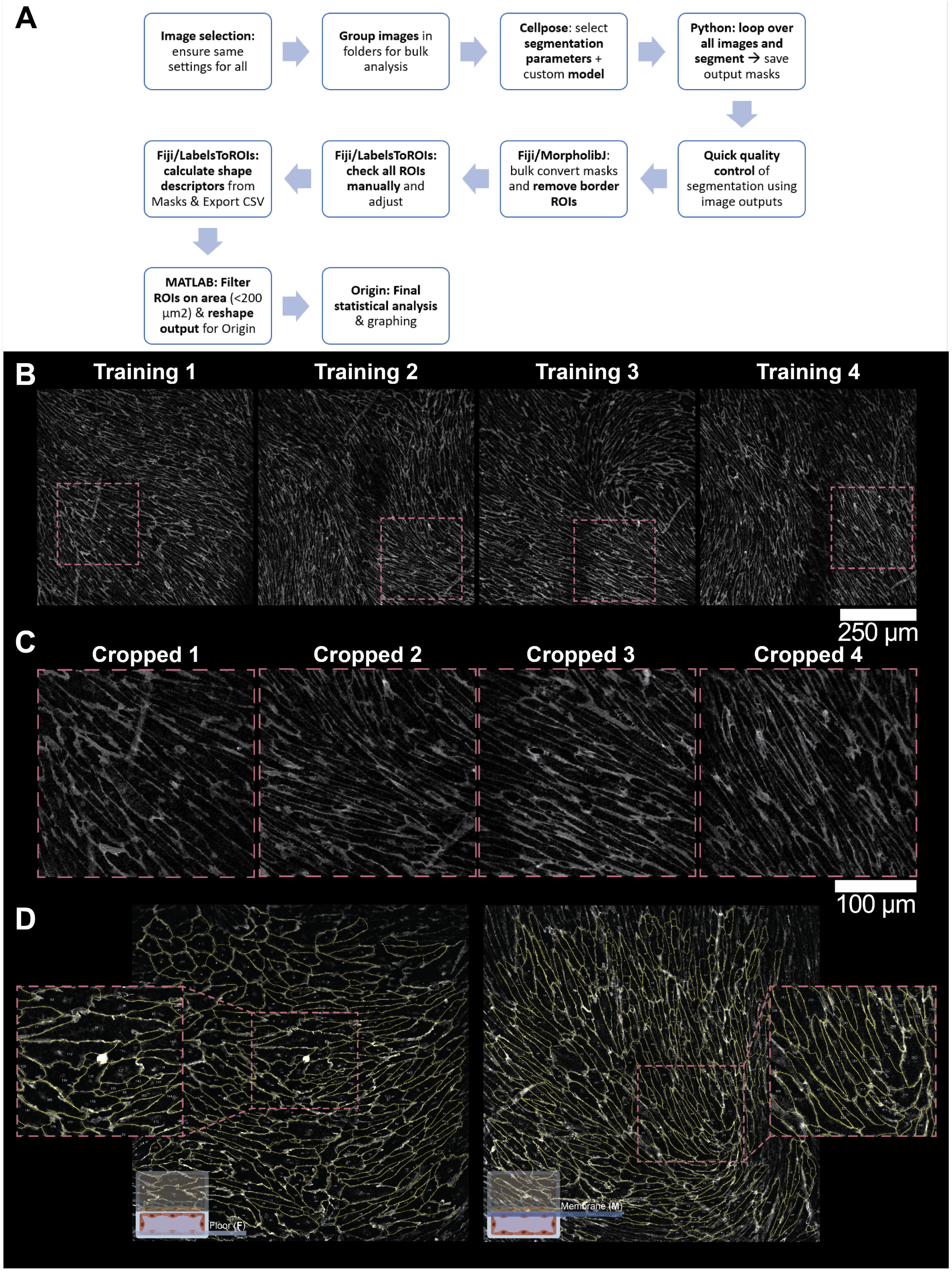

Figure S10. Cellpose workflow and example output for VE-cadherin images. A) Complete Cellpose workflow, building upon previous work by others.[63] **B)** Training dataset (subset of the complete experimental dataset) representing the morphologies found in this work. Red dashed squares represent the location at which the cropped images in (C) were taken. **C)** Cropped versions of the training dataset in (B). **D)** Example of segmentation (LabelsToROIs output) for floor-attached cells (*left*) and membrane-attached cells (*right*) at Day 10 in a triple co-culture sample. Note that the ROI borders (yellow lines) follow the VE-cadherin signal as expected.

Table S1. Antibodies used for immunofluorescence staining in this work.

Target	Host	Clone	Concentration (μg/mL)	Block buffer	Supplier	Cat. No.
Cadherin 11	Rabbit	Polyclonal	2.5	5% BSA in 0.1% PBST	Invitrogen	71-7600
CD90/THY1	Mouse	5E10	5	8% Goat Serum and 2% BSA in PBS	Invitrogen eBioscience™	14-0909-82
CD68	Mouse	KP1	10	5% BSA in 0.1% PBST	Abcam	ab955
CD163	Rabbit	EPR19518	7	5% BSA in 0.1% PBST	Abcam	ab182422
VE-cadherin	Mouse	F-8	1	5% BSA in 0.1% PBST	Santa Cruz Biotechnologies	SC-9989
ICAM-1	Mouse	11C81	5	5% BSA in 0.1% PBST	R&D Systems	BBA3

Table S2. Kruskal-Wallis ANOVA output for endothelial cell Area based on VE-cadherin segmentation using Cellpose. Highlights, yellow: comparisons within the same day between floor and memb(rane); green: comparisons for floor-floor and memb-memb over different time points. Sig: significance based on a significance threshold of Prob < 0.05.

	Comparison	Mean Rank Diff	Z	Prob	Sig
Area	T3Floor T3Memb	511,51809	4,62037	<0.0001	1
	T3Floor T5Floor	-781,83692	-7,10634	<0.0001	1
	T3Floor T5Memb	-541,11648	-5,17637	<0.0001	1
	T3Floor T10Floor	-508,49322	-5,78613	<0.0001	1
	T3Floor T10Memb	358,90723	4,00298	9,38E-04	1
	T3Memb T5Floor	-1293,35501	-12,2227	<0.0001	1
	T3Memb T5Memb	-1052,63457	-10,5156	<0.0001	1
	T3Memb T10Floor	-1020,01131	-12,3551	<0.0001	1
	T3Memb T10Memb	-152,61087	-1,80714	1	0
	T5Floor T5Memb	240,72044	2,42322	0,23075	0
	T5Floor T10Floor	273,3437	3,34853	0,01219	1
	T5Floor T10Memb	1140,74414	13,65462	<0.0001	1
	T5Memb T10Floor	32,62326	0,44041	1	0
	T5Memb T10Memb	900,0237	11,815	<0.0001	1
	T10Floor T10Memb	867,40044	17,01695	<0.0001	1

Table S3. Kruskal-Wallis ANOVA output for endothelial cell Feret Angle based on VE-cadherin segmentation using Cellpose. Highlights, yellow: comparisons within the same day between floor and memb(rane); green: comparisons for floor-floor and memb-memb over different time points. Sig: significance based on a significance threshold of Prob < 0.05.

	**Comparison**	**Mean Rank Diff**	**Z**	**Prob**	**Sig**
**Feret Angle**	T3Floor T3Memb	-8,0272	-0,07251	1	0
	T3Floor T5Floor	-125,20786	-1,13805	1	0
	T3Floor T5Memb	-73,18445	-0,70009	1	0
	T3Floor T10Floor	311,59632	3,54565	0,00587	1
	T3Floor T10Memb	-907,24599	-10,1187	<0.0001	1
	T3Memb T5Floor	-117,18066	-1,1074	1	0
	T3Memb T5Memb	-65,15725	-0,65091	1	0
	T3Memb T10Floor	319,62352	3,87149	0,00162	1
	T3Memb T10Memb	-899,21879	-10,6481	<0.0001	1
	T5Floor T5Memb	52,02341	0,5237	1	0
	T5Floor T10Floor	436,80419	5,35096	<0.0001	1
	T5Floor T10Memb	-782,03813	-9,36093	<0.0001	1
	T5Memb T10Floor	384,78078	5,1945	<0.0001	1
	T5Memb T10Memb	-834,06154	-10,9491	<0.0001	1
	T10Floor T10Memb	-1218,84231	-23,9117	<0.0001	1

Table S4. Kruskal-Wallis ANOVA output for endothelial cell Aspect Ratio based on VE-cadherin segmentation using Cellpose. Highlights, yellow: comparisons within the same day between floor and memb(rane); green: comparisons for floor-floor and memb-memb over different time points. Sig: significance based on a significance threshold of Prob < 0.05.

	Comparison	Mean Rank Diff	Z	Prob	Sig
Aspect Ratio	T3Floor T3Memb	-311,05502	-2,80966	0,07439	0
	T3Floor T5Floor	-235,50888	-2,14061	0,48458	0
	T3Floor T5Memb	-562,77206	-5,38353	<0.0001	1
	T3Floor T10Floor	-1299,05321	-14,7819	<0.0001	1
	T3Floor T10Memb	-2624,80595	-29,2751	<0.0001	1
	T3Memb T5Floor	75,54613	0,71394	1	0
	T3Memb T5Memb	-251,71705	-2,5146	0,17875	0
	T3Memb T10Floor	-987,99819	-11,9673	<0.0001	1
	T3Memb T10Memb	-2313,75093	-27,3982	<0.0001	1
	T5Floor T5Memb	-327,26318	-3,29441	0,01479	1
	T5Floor T10Floor	-1063,54433	-13,0287	<0.0001	1
	T5Floor T10Memb	-2389,29707	-28,5997	<0.0001	1
	T5Memb T10Floor	-736,28115	-9,93972	<0.0001	1
	T5Memb T10Memb	-2062,03388	-27,0692	<0.0001	1
	T10Floor T10Memb	-1325,75274	-26,0091	<0.0001	1
